# Sumoylated NHR-25/NR5A Regulates Cell Fate during *C. elegans* Vulval Development

**DOI:** 10.1371/journal.pgen.1003992

**Published:** 2013-12-12

**Authors:** Jordan D. Ward, Nagagireesh Bojanala, Teresita Bernal, Kaveh Ashrafi, Masako Asahina, Keith R. Yamamoto

**Affiliations:** 1 Department of Cellular and Molecular Pharmacology, University of California San Francisco, San Francisco, California, United States of America; 2 Institute of Parasitology, Biology Centre ASCR, Ceske Budejovice, Czech Republic; 3 University of South Bohemia, Ceske Budejovice, Czech Republic; 4 Department of Physiology, University of California San Francisco, San Francisco, California, United States of America; University of Texas Southwestern Medical Center, United States of America

## Abstract

Individual metazoan transcription factors (TFs) regulate distinct sets of genes depending on cell type and developmental or physiological context. The precise mechanisms by which regulatory information from ligands, genomic sequence elements, co-factors, and post-translational modifications are integrated by TFs remain challenging questions. Here, we examine how a single regulatory input, sumoylation, differentially modulates the activity of a conserved *C. elegans* nuclear hormone receptor, NHR-25, in different cell types. Through a combination of yeast two-hybrid analysis and *in vitro* biochemistry we identified the single *C. elegans* SUMO (SMO-1) as an NHR-25 interacting protein, and showed that NHR-25 is sumoylated on at least four lysines. Some of the sumoylation acceptor sites are in common with those of the NHR-25 mammalian orthologs SF-1 and LRH-1, demonstrating that sumoylation has been strongly conserved within the NR5A family. We showed that NHR-25 bound canonical SF-1 binding sequences to regulate transcription, and that NHR-25 activity was enhanced *in vivo* upon loss of sumoylation. Knockdown of *smo-1* mimicked NHR-25 overexpression with respect to maintenance of the 3° cell fate in vulval precursor cells (VPCs) during development. Importantly, however, overexpression of unsumoylatable alleles of NHR-25 revealed that NHR-25 sumoylation is critical for maintaining 3° cell fate. Moreover, SUMO also conferred formation of a developmental time-dependent NHR-25 concentration gradient across the VPCs. That is, accumulation of GFP-tagged NHR-25 was uniform across VPCs at the beginning of development, but as cells began dividing, a *smo-1*-dependent NHR-25 gradient formed with highest levels in 1° fated VPCs, intermediate levels in 2° fated VPCs, and low levels in 3° fated VPCs. We conclude that sumoylation operates at multiple levels to affect NHR-25 activity in a highly coordinated spatial and temporal manner.

## Author Summary

Animals precisely control when and where genes are expressed; failure to do so can cause severe developmental defects and pathology. Transcription factors must display extraordinary functional flexibility, controlling very different sets of genes in different cell and tissue types. To do so, they integrate information from signaling pathways, chromatin, and cofactors to ensure that the correct ensemble of genes is orchestrated in any given context. The number of regulatory inputs, and the complex physiology and large numbers of cell and tissue types in most experimentally tractable metazoans have rendered combinatorial regulation of transcription nearly impenetrable. We used the powerful genetics and simple biology of the model nematode, *C. elegans*, to examine how a single post-translational modification (sumoylation) affected the activity of a conserved TF (NHR-25) in different cell types during animal development. Our work suggests that sumoylation constrains NHR-25 activity in order to maintain proper cell fate during development of the reproductive organ.

## Introduction

Tissue-specific and cell type-specific transcriptional networks underlie virtually every aspect of metazoan development and homeostasis. Single TFs, operating within gene-specific regulatory complexes, govern distinct gene regulatory networks in different cells and tissues; thus, combinatorial regulation underpins tissue- and cell type-specific transcription. Determining the precise mechanisms whereby such specificity arises and how networks nevertheless remain flexible in responding to environmental and physiological fluctuations is an interesting challenge. TFs integrate signaling information from co-factors, chromatin, post-translational modifications, and, in the case of nuclear hormone receptors, small molecule ligands, to establish transcription networks of remarkable complexity.

Here, we approach this problem by studying a covalent modification of a nuclear hormone receptor (NHR) in *C. elegans*, a simple metazoan with powerful genetic tools, a compact genome, and an invariant cell lineage leading to well-defined tissues. NHRs are DNA-binding TFs characterized by a zinc-finger DNA binding domain (DBD) and a structurally conserved ligand binding domain (LBD) [1]. The genome of *C. elegans* encodes 284 NHRs while humans only have 48 NHRs [1]. Of the 284 NHRs, 269 evolved from an HNF4α-like gene [2], and 15 have clear orthologs in other species. NHR-25 is the single *C. elegans* ortholog of vertebrate SF-1/NR5A1and LRH-1/NR5A2, and arthropod Ftz-F1 and fulfills many criteria for the study of tissue-specific transcriptional networks [1]. NHR-25 is broadly expressed in embryos and in epithelial cells throughout development [3], [4]. It is involved in a range of biological functions such as molting [3]–[5], heterochrony [6], and organogenesis [7]. Furthermore, both NHR-25 and its vertebrate orthologs regulate similar processes. SF-1 and NHR-25 promote gonadal development and fertility [8], [9], while NHR-25 and LRH-1 both play roles in embryonic development and fat metabolism [4], [10]–[12]. The pleiotropic phenotypes seen following RNAi or mutation of *nhr-25* highlight the broad roles of the receptor, and its genetic interaction with numerous signaling pathways (β-catenin, Hox, heterochronic network) [6]–[8] make it an excellent model to study combinatorial gene regulation by NHRs.

SUMO (small ubiquitin-like modifier) proteins serve as post-translational modifiers and are related to but distinct from ubiquitin [13]; we show here that NHR-25 is sumoylated. Sumoylation uses similar enzymology as ubiquitination to conjugate the SUMO protein onto substrate lysines [13]. Briefly, SUMO is produced as an inactive precursor. A SUMO protease activates SUMO by cleaving residues off the C-terminus to expose a di-glycine [13]. A heterodimeric E1 protein consisting of UBA2 and AOS1 forms a thioester bond with the exposed diglycine and then transfers SUMO to an E2 enzyme (UBC9), also through a thioester bond [14]. The E2 enzyme then either directly conjugates SUMO onto a target lysine, or an E3 ligase can enhance the rate of sumoylation; that is, unlike in ubiquitination, E3 ligases are not always required. Like many post-translational modifications, sumoylation is reversible and highly dynamic. The same SUMO protease that initially activated SUMO cleaves the isopeptide linkage that covalently attaches SUMO to the target protein [14]. Indeed, global failure to remove SUMO from substrates compromises viability in mice and *S. pombe* [15], [16].

The extent of sumoylation of a given target can be regulated by varying the expression, localization, stability or activity of components of the sumoylation machinery in response to external and internal cellular cues [14]. SUMO-regulated processes include nuclear-cytosolic transport, DNA repair, transcriptional regulation, chromosome segregation and many others [14]. For example, sumoylation of the glucocorticoid receptor prevents synergy between two GR dimers bound at a single response element [17]. In this sense, SUMO is analogous to the small hydrophobic hormones and metabolites that serve as noncovalent ligands for nuclear receptors, except it associates both covalently and non-covalently with its targets. Sumoylation modulates the activities of multiple classes of cellular proteins, such as transcriptional regulators, DNA replication factors and chromatin modifiers.

Elucidating how a single nematode NHR integrates cellular signals to regulate specific genes in distinct tissues will advance our understanding of metazoan transcription networks. To this end, we examined how sumoylation regulates the *C. elegans* nuclear hormone receptor NHR-25, and the physiological relevance of this nuclear hormone receptor-SUMO interaction. Using a combination of genetics, cell biology, and *in vitro* biochemistry we sought to understand how signaling through sumoylation impacts NHR-25's role in animal development, and how sumoylation affects the NHR-25 transcriptional network.

## Results

### NHR-25 physically interacts with SMO-1

**Figure 1:**
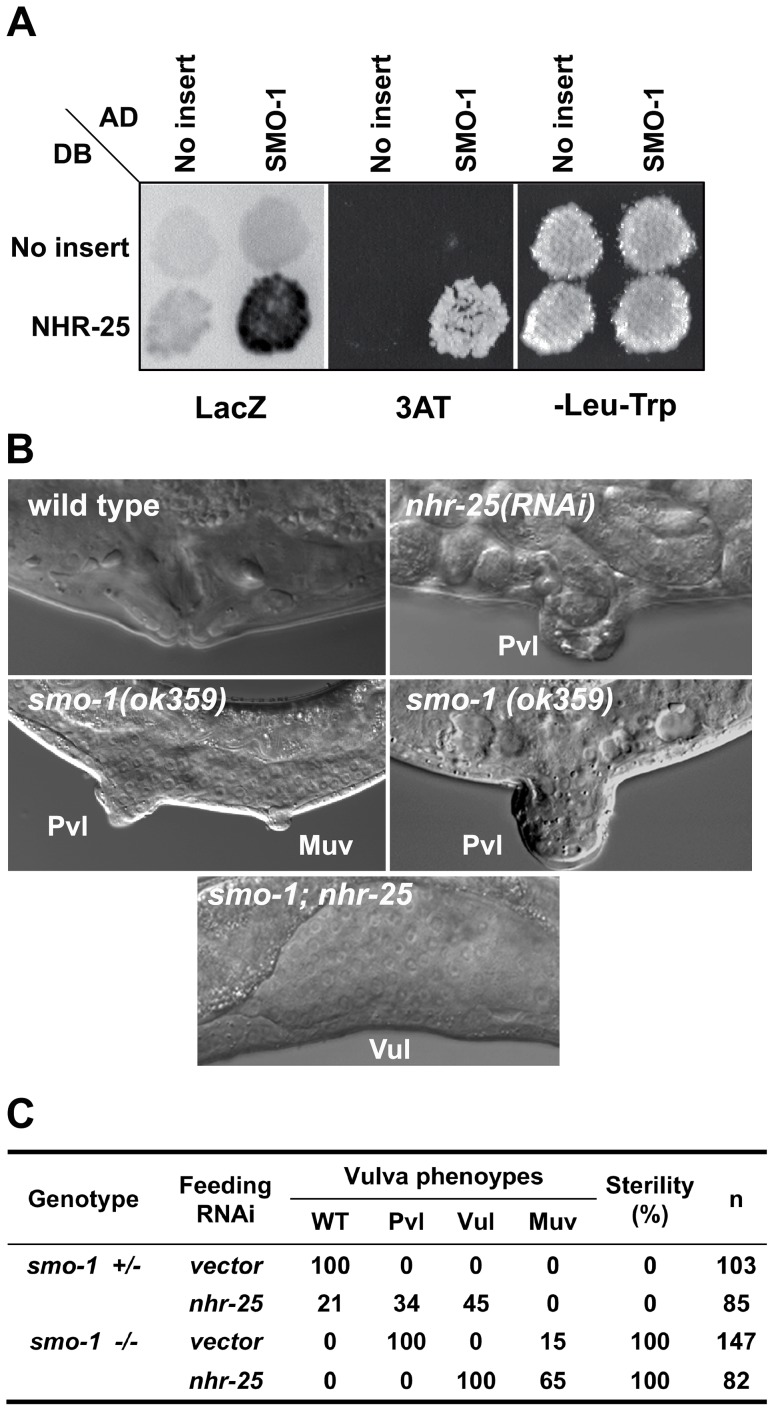
SMO-1 and NHR-25 physically and genetically interact. (A) NHR-25 fused to the Gal4 DNA binding domain (DB) interacted with wild type (WT) SMO-1 fused to the Gal4 activation domain (AD). No interaction was seen with empty vector (No insert). β-galactosidase (LacZ) and HIS3 (3AT; 3-aminotriazole) reporters were assayed, and yeast viability was confirmed by growth on a plate lacking leucine and tryptophan (-Leu-Trp). (B) DIC microscopy examining vulval morphology in animals of the indicated genotype. Characteristic protruding vulvae (Pvl) seen in *nhr-25(RNAi)* and *smo-1(ok359)* animals are indicated, as is the low penetrance multivulva phenotype (Muv) of *smo-1(lf)* animals. RNAi inactivation of *nhr-25* in a *smo-1(ok359)* mutant resulted in vulvaless (Vul) animals. (C) Table providing scoring of the Pvl, Vul, Muv, and sterility phenotypes of the indicated genotypes. n = number of animals scored.

We identified an interaction between NHR-25 and the single *C. elegans* SUMO homolog (SMO-1) in a genome-wide Y2H screen using the normalized AD-Orfeome library, which contains 11,984 of the predicted 20,800 *C. elegans* open reading frames [18]. SMO-1 was the strongest interactor in the screen on the basis of two selection criteria, staining for β-galactosidase activity and growth on media containing 3-aminotriazole (Figure 1A). To assess the selectivity of the SMO-1–NHR-25 interaction, we tested pairwise combinations of SMO-1 with full-length NHR-25, an NHR-25 isoform β that lacks the DNA-binding domain, and each of seven additional NHRs: NHR-2, NHR-10, NHR-31, NHR-91, NHR-105, FAX-1, and ODR-1 (Figure S1A). The NHR-25-SMO-1 interaction proved to be selective, as SMO-1 failed to bind the other NHRs tested. NHR-25 also interacted with the GCNF homolog, NHR-91 (Figure S1A).

### *nhr-25* and *smo-1* genetically interact during vulval development

**Figure 2:**
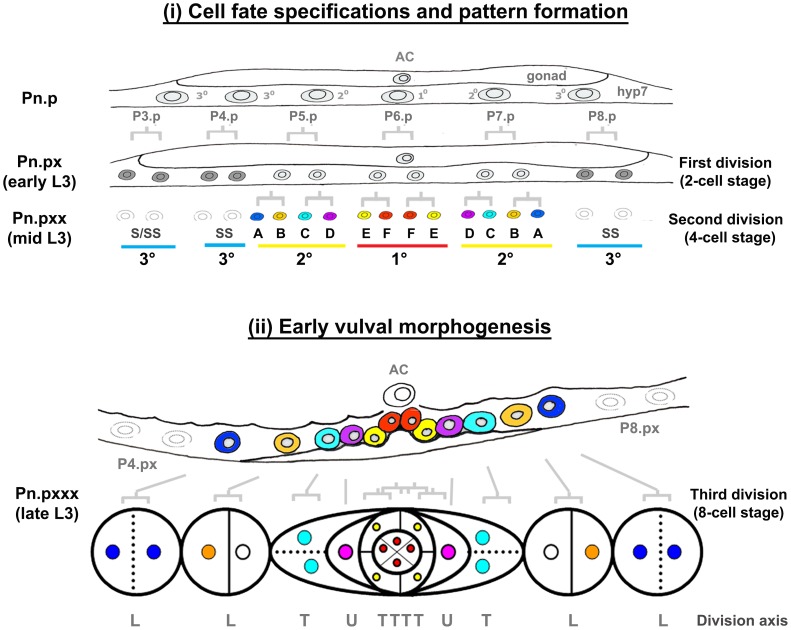
Vulval morphogenesis. The fully formed vulva of *C. elegans* is generated post-embryonically from cell divisions of three vulval precursor cells (VPCs). These three VPCs are denoted as P5.p, P6.p and P7.p and undergo a series of stereotyped divisions producing 22 cells. Cells arising from the P6.p precursor are designated as having the primary (1°) fate, while those arising from P5.p and P7.p precursors are designated as having the secondary (2°) fate. 2° cells generate vulA-D cells and 1° cells generate vulE and vulF. In early to mid L3 larvae, the proximity to a gonadal cell known as the anchor cell (AC) initiates vulval patterning by secretion of LIN-3/EGF [64]. The closest VPC to the AC (P6.p) receives the highest LIN-3/EGF dose, which activates LET-60/Ras signaling in P6.p [65]–[68], prompting it to adopt a 1° fate. This EGF-Ras signaling also induces P6.p to express the Notch ligand. The moderate level of LIN-3 received by neighboring P5.p and P7.p cells combined with lateral inhibition through the Notch pathway, induces P5.p and P7.p cells to adopt a 2° fate. In the L3 larval stage, the 1° and 2° cell lineages divide three times, and undergo a coordinated series of migrations and fusions during morphogenesis to complete vulval development [38]. Three VPCs (P3.p, P4.p and P8.p) normally adopt a 3° fate, which means that they divide once and fuse into an epidermal syncytial cell called hyp7, with the exception of P3.p of which about 50% of the lineage fuses without division (designated as S). Syncytial fate is designated S or SS in the figure. The pattern of cell division axes are depicted as L (longitudinal), T (transverse) and U (undivided).

SMO-1 was an enticing NHR-25 interacting partner to pursue. SUMO in *C. elegans* and other eukaryotes regulates TFs and chromatin, thus is well positioned to impact NHR-25 gene regulatory networks. Furthermore, spatial and temporal expression patterns of *smo-1* and *nhr-25* during development largely overlap [3], [4], [19]. SUMO interacts with the mammalian homologs of NHR-25, suggesting that the interaction is likely evolutionarily conserved [20], [21]. Among its many phenotypes, *smo-1* loss-of-function (*lf*) mutants display a fully penetrant protruding vulva (Pvl) phenotype, reflecting disconnection of the vulva from the uterus [19] (Figure 1B, C). *smo-1* RNAi or mutation also cause low penetrance of ectopic induction of vulval cells, which can generate non-functional vulval-like structures known as multivulva (Muv) [22] (Figure 1B, C). Similar to *smo-1* mutants, *nhr-25* reduction-of-function leads to a Pvl phenotype, but does not cause Muv [7]. This *nhr-25* Pvl phenotype results from defects in cell cycle progression, aberrant division axes of 1° and 2° cell lineages, and altered vulval cell migration (Table 1, Figure 2, Bojanala *et al.*, manuscript in preparation). Because at an earlier stage NHR-25 is also necessary for establishing the anchor cell (AC) [8], which secretes the EGF signal that initiates vulval precursor cell (VPC) patterning, our RNAi treatments were timed to allow AC formation and examination of the effect of *nhr-25* depletion on later developmental events.

**Table 1:** Vulva cell lineage analyses in *nhr-25(RNAi)* and *smo-1(lf)* animals.

Genotype	Pn.p lineages						
	P3.p	P4.p	P5.p	P6.p	P7.p	P8.p	n
wild type	S	SS	LLTU	TTTT	UTLL	SS	4
	SS	SS	LLTU	TTTT	UTLL	SS	3
*smo-1(ok359)*	S	SS	LLTU	TTTT	UTLL	SS	3
	SS	SS	LLTU	TTTT	UTLL	SS	2
*nhr-25(RNAi)*	S	SS	LLTU	TTTT	UTLL	SS	2
	SS	SS	LLUU	LUUL	UULL	SS	2
	SS	SS	LLUU	LUUL	ULLL	SS	1
*smo-1(ok359);*	S	SS	UUUU	UUUU	UUUU	SS	3
*nhr-25(RNAi)*	SS	SS	UUUU	UUUU	UUUU	SS	4
	S	S LU	UUUU	LUUL	UUUU	SS	1
	SS	SS	LLUU	UUUU	UUUU	SS	1
	SS	UU D	LLUU	UUUU	UU S	LU S	1
	S	UULL	UUUU	UUUU	UUUU	SS	1
	UULU	UUUU	UUUU	UUUU	UUUU	LUUU	1
	SS	UUUU	UUUU	UUUU	UUUU	SS	1
	SS	S LU	UUUU	UUUU	UUUU	SS	1
	SS	UUUL	UUUU	UUUU	UUUU	UUUU	1

S- syncytial fate; L- longitudinal; T – transverse; D-undetermined and U- undivided cell.

When *smo-1* and *nhr-25* were simultaneously inactivated, animals exhibited a fully penetrant vulvaless (Vul) phenotype and an exacerbated Muv phenotype (Figure 1B, C). The ectopically induced vulval cells expressed an *egl-17::YFP* reporter, indicating that 3°-fated cells aberrantly adopted 1° and 2° fates in these animals (Figure S2B). This *egl-17::YFP* reporter allowed us to monitor 1°/2° fate induction despite the cell division arrest phenotypes of *nhr-25(RNAi)* and *smo-1(lf);nhr-25(RNAi)* animals. Lineage analyses showed that following simultaneous inactivation of both *smo-1* and *nhr-25*, daughters of all VPCs normally responsible for vulva formation, (P5.p, P6.p and P7.p) failed to undergo the third round of vulval cell division (Table 1) resulting in premature cell division arrest and the Vul phenotype. Although P5.p, P6.p and P7.p VPCs were induced, the execution of 2° fate was abnormal: in both *smo-1(ok359)* and *smo-1(ok359);nhr-25(RNAi)* backgrounds, the expression of the 1° marker, *egl-17::YFP* exhibited ectopically high expression in P5.p and/or P7.p (Figure S2A) at the 4-cell stage. Moreover, in *smo-1;nhr-25(RNAi)* animals, the P(3,4,8).p cell, which normally divides only once and fuses into the hypodermal syncytium, kept dividing (Table 1). This continued division enhanced the Muv induction phenotype seen in *smo-1* mutants. Thus, reduction of SMO-1 activity enhanced cell division defects in 1° and 2° *nhr-25* mutant VPCs, while reduction of NHR-25 activity enhanced the *smo-1* mutant Muv phenotype in 3° fated cells.

### SMO-1 binds NHR-25 covalently and non-covalently

**Figure 3:**
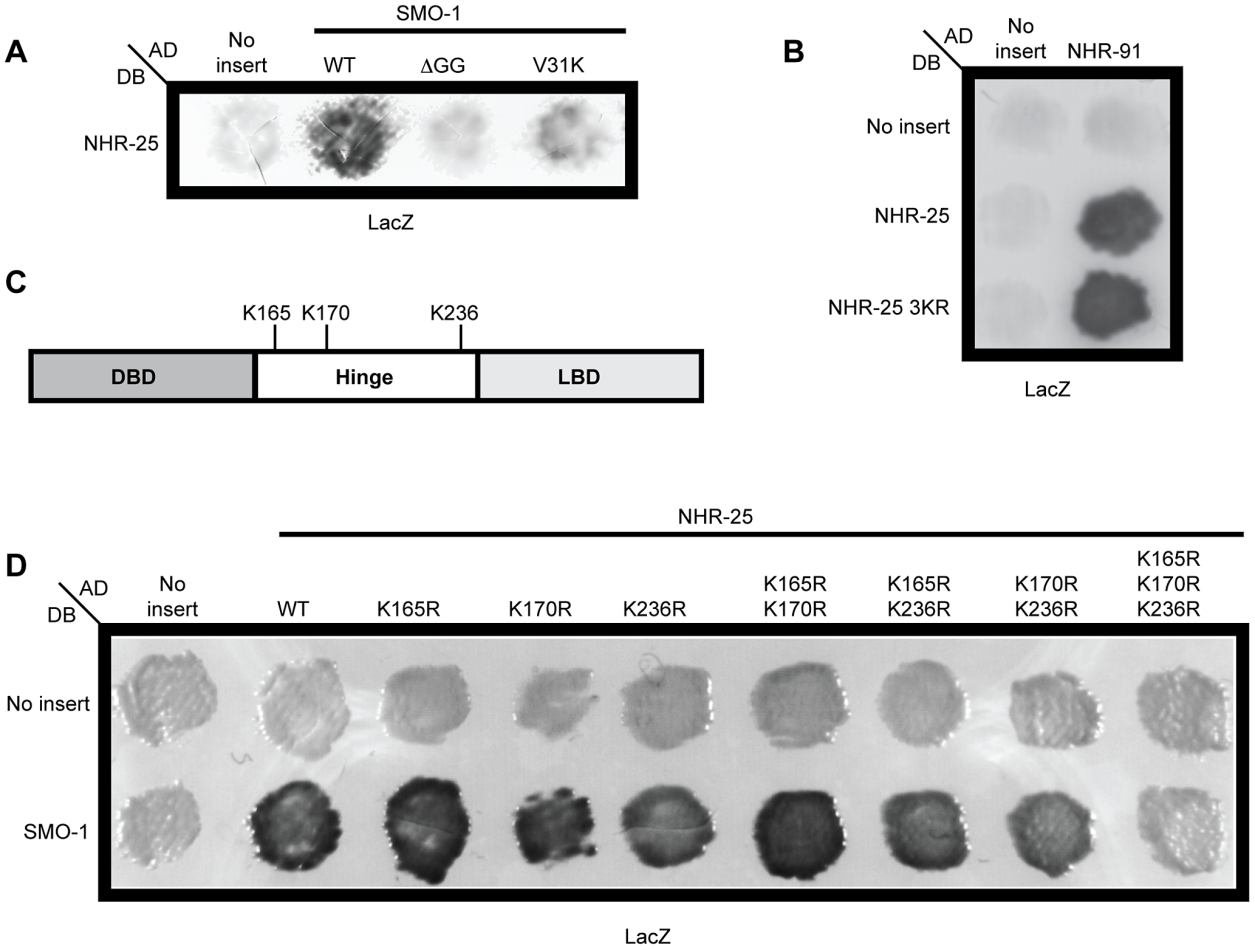
Three lysines in NHR-25 are necessary for the interaction with SMO-1. (A) NHR-25 fused to the Gal4 DNA binding domain (DB) interacted with wild type (WT) SMO-1 fused to the Gal4 activation domain (AD). No interaction was seen with empty vector (No insert), SMO-1 with the terminal di-glycine residues deleted (ΔGG), or SMO-1 with a β-sheet mutation (V31K). (B) The NHR-25 3KR (K165R K170R K236R) allele specifically blocked interaction with SMO-1, as both NHR-25 and NHR-25 3KR interacted with NHR-91. (C) Schematic of NHR-25 domain structure illustrating the DNA binding domain (DBD), hinge region, and ligand binding domain (LBD). The candidate SUMO acceptor lysines (K165, K170, K236) are indicated. (D) Mutating the indicated SUMO acceptor lysines to arginine in NHR-25 only abolished the interaction when all three were mutated (K165R K170R K236R). We note the non-reciprocality of our Y2H interactions: DB-NHR-25 interacted with AD-SMO-1 and AD-NHR-25 interacted with DB-NHR-91. Switching the Gal4 domains did not result in an interaction, as sometimes occurs in Y2H interactions [69]. β-galactosidase (LacZ) reporters were assayed in A, B, and D.

NHR-25 and SMO-1 interact physically in Y2H assays and genetically *in vivo*, consistent with their overlapping expression patterns [4], [19]. Furthermore, the mammalian NHR-25 homologs are sumoylated, suggesting that SMO-1-NHR-25 interactions are conserved and physiologically important. Y2H interactions with SUMO can reflect non-covalent binding, or covalent sumoylation where the SUMO protein is coupled onto the substrate through an isopeptide bond. These two possibilities can be distinguished genetically. Mutations in the β-sheet of SUMO interfere with non-covalent binding, whereas deletion of the terminal di-glycine in SUMO selectively compromises covalent sumoylation [23]. As can be seen in Figure 3A, deletion of the terminal di-glycine residues of SMO-1 (ΔGG) completely abrogated the interaction with NHR-25. The SMO-1 V31K mutation predicted to disrupt the conserved β-sheet of SMO-1 hampered the Y2H interaction between NHR-25 and SMO-1, although not as severely as the SMO-1 ΔGG mutation (Figure 3A). These findings are similar to those with DNA thymine glycosylase and the Daxx transcriptional corepressor, both of which bind SUMO non-covalently and are also sumoylated [24], [25]. The V31K β-sheet mutant was competent to bind the *C. elegans* SUMO E2 enzyme, UBC-9, confirming its correct folding (Figure S3A). Together, these results suggested that NHR-25 is both sumoylated and binds SMO-1 non-covalently; conceivably, the two modes of interaction confer distinct regulatory outcomes.

### Three lysines in the hinge region of NHR-25 are required for sumoylation

As our Y2H data suggested that NHR-25 was sumoylated, we identified candidate sumoylation sites within NHR-25 using the SUMOsp2.0 prediction program [26]. The sumoylation consensus motif is ψ-K-X-D/E, where ψ is any hydrophobic amino acid, K is the lysine conjugated to SUMO, X is any amino acid, and D or E is an acidic residue [14]. Three high scoring sites reside in the hinge region of the protein: two are proximal to the DBD (K165 and K170) and one (K236) is near the LBD (Figure 3C). We mutated these sites, conservatively converting the putative SUMO acceptor lysine residues to arginine to block sumoylation. Single mutation of any of the three candidate lysines had no apparent effect on the NHR-25 interaction with SMO-1 in Y2H assays, whereas the three double mutants had modest effects, and the NHR-25 3KR triple mutant (K165R K170R K236R) abrogated binding (Figure 3D). A fourth candidate sumoylation site (K84) located in the DBD was completely dispensable for the Y2H interaction (data not shown). To verify that the 3KR mutations blocked the interaction with SMO-1 specifically, rather than causing NHR-25 misfolding or degradation, we confirmed that NHR-25 3KR retained the capacity to bind NHR-91 (Figures S1, Figure 3B). These data suggested that either non-covalent binding is dispensable for the SMO-1-NHR-25 interaction and that this was a rare case in which the SUMO β-sheet mutation impaired sumoylation, or that the three lysines in NHR-25 were important for both the covalent and non-covalent interaction with SMO-1.

**Figure 4:**
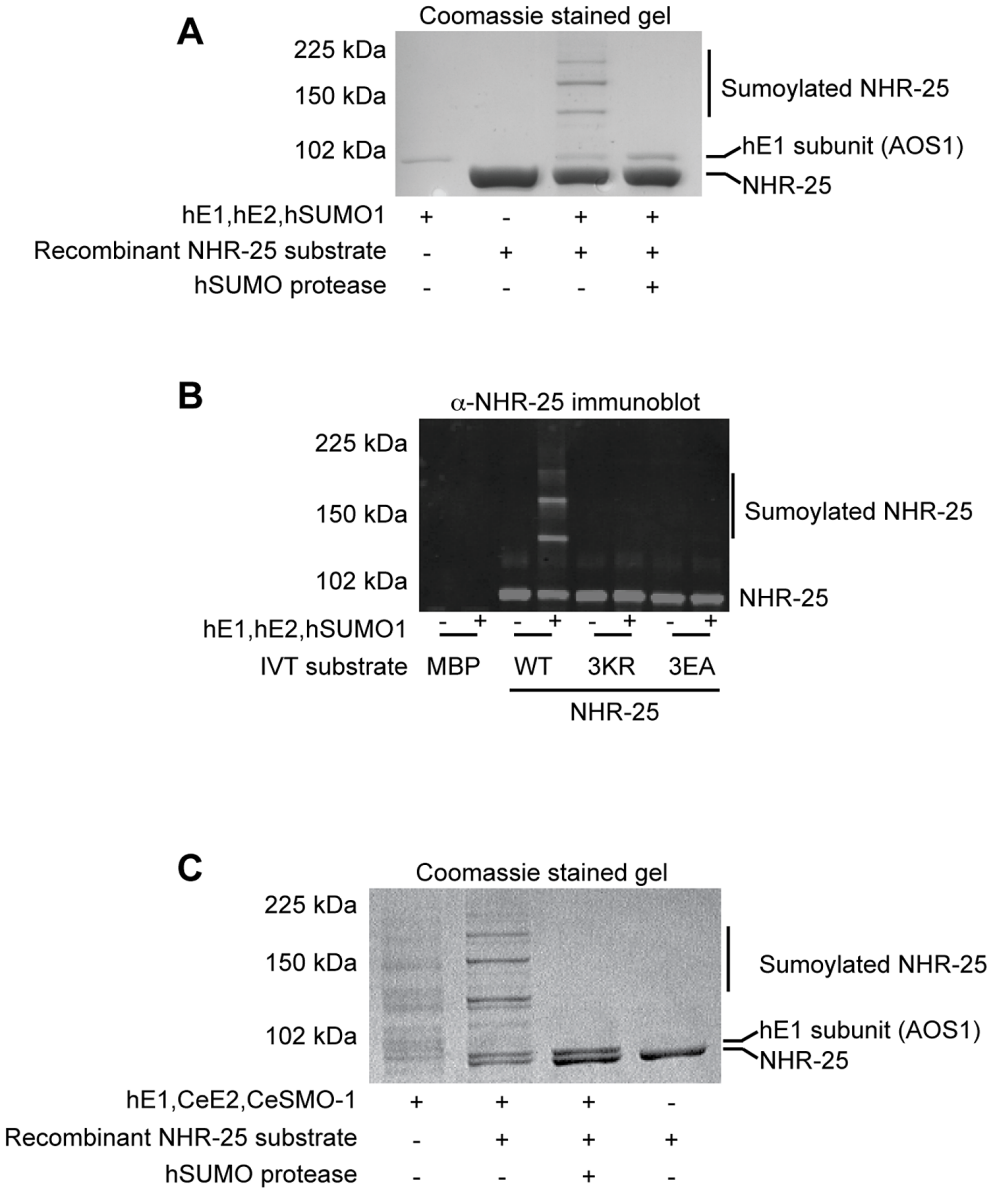
*In vitro* sumoylation of NHR-25. *In vitro* sumoylation reactions were resolved by SDS-PAGE and analyzed by either Coomassie staining (A,C) or immunoblotting with anti-NHR-25 antibody (B). (A and B) used recombinant human sumoylation enzymes (hE1, hE2, hSUMO1, hSENP1 SUMO protease), (C) used recombinant *C. elegans* CeUBC-9 and CeSMO-1 with hE1 and hSENP1. Substrates were recombinant 6×His-MBP-NHR-25 (amino acids 161–541; A,C), and the same construct *in vitro* transcribed and translated (B). In (B) an MBP control was *in vitro* transcribed and translated, as were the NHR-25 alleles 3KR (K165 K170R K236R) and 3EA(E167A E172A E238A). The positions of NHR-25, sumoylated NHR-25 and AOS1 (part of E1 heterodimer) are indicated. Size markers in kilodaltons (kDa) are provided.

To ensure that our Y2H results indeed reflected NHR-25 sumoylation, we turned to *in vitro* sumoylation assays. As both human and *C. elegans* sumoylation enzymes were used in these experiments, we distinguish them with prefixes “h” and “Ce”. As a positive control, we expressed and purified recombinant hE1, hUBC9, hSUMO1, and hSENP1 from *E. coli*. We also purified a recombinant partial hinge-LBD fragment of mouse SF-1 from *E. coli*; this fragment contains a single sumoylation site in the hinge region. SF-1 is a vertebrate ortholog of NHR-25 and the fragment that we used is a robust sumoylation substrate (Figure S4A) [27]. We then purified an N-terminally hexahistidine-Maltose Binding Protein (6×His-MBP) tagged fragment of NHR-25 (amino acids 161–541) containing most of the hinge region and ligand-binding domain, including all three candidate SUMO acceptor lysines. Coomassie staining and immunoblotting revealed three slower-migrating species, which were collapsed by the addition of the SUMO protease, hSENP1 (Figure 4A, S5A). We detected sumoylation of the same 6×HisMBP-NHR-25 fragment when it was expressed in rabbit reticulocyte lysates, followed by incubation with hE1, hE2 and hSUMO1 (Figure 4B).

We further tested NHR-25 substrates containing two (2KR; K170R K236R) or three arginine substitutions (NHR-25 3KR). When only one predicted acceptor lysine was available (2KR), we detected a single dominant sumoylated species, whereas for NHR-25 3KR, sumoylation was abrogated (Figure S5B). We performed sumoylation reactions on *in vitro* transcribed and translated wild type NHR-25, NHR-25 3KR, and NHR-25 3EA. In NHR-25 3EA (E167A E172A E238A) the acidic glutamic acid residues within the three consensus sumoylation sites were mutated to alanine. NHR-25 3EA leaves the acceptor lysines available, but is predicted to inhibit sumoylation by impairing interaction with UBC9. While wild type NHR-25 was clearly sumoylated, the 3EA mutation severely impaired sumoylation (Figure 4B).

When sumoylation reaction times were extended 5–20 fold, additional species of sumoylated NHR-25 were generated (Figure S6A). These species could reflect sumoylation of NHR-25 on other sites or formation of hSUMO1 chains. To distinguish between these possibilities, we used methyl-hSUMO1, which can be conjugated onto a substrate lysine, but chain formation is blocked by methylation. Long incubations with methyl-hSUMO1 resulted in only three sumoylated NHR-25 bands, as determined by NHR-25 immunoblotting, indicating that there are indeed only the three major acceptor lysines (Figure S6A). hSUMO2, which readily forms polySUMO chains, was included as a control in this experiment. Even with extended incubation times, we observed only three dominant sumoylated forms of NHR-25, suggesting that additional bands in reactions using hSUMO1 or CeSMO-1 reflect inefficient chaining. We conclude that NHR-25 is sumoylated *in vitro* on three lysines and that *C. elegans* SMO-1 does not readily form polySUMO chains, unlike yeast SMT3 and mammalian SUMO2.

### Biochemical characterization of *C. elegans* UBC-9 and SMO-1

All studies of *C. elegans* sumoylation to date have used hE1, hUBC9, and hSUMO proteins [19], [28], [29]. We purified recombinant CeE1, CeUBC-9 and CeSMO-1 from *E. coli* and tested their activity in *in vitro* sumoylation assays. Our CeE1 preparation was inactive, but was effectively substituted by hE1. Under those conditions, our CeUBC-9 and CeSMO-1 catalyzed sumoylation of the SF-1 hinge-LBD fragment (Figure S4B). Similar to hUBC9 and hSUMO1, recombinant CeUBC-9 and CeSMO-1 yielded three sumoylated species using the 6×His-MBP-NHR-25 substrate (Figure 4C, S4C).

To determine the kinetics of the three SUMO modifications of NHR-25, we performed a time course of standard sumoylation reactions with hUBC9/CeUBC-9 and hSUMO1/CeSMO-1 proteins. In both cases, we detected a single band by 15 minutes, followed by two and then three sumoylated species as the reaction progressed (Figure S6B–E). These data imply that the three sumoylation sites are modified sequentially, in a particular order.

All of our reactions were performed without addition of an E3 ligase. The high efficiency of SF-1 sumoylation in the absence of E3 ligase is in part due to a direct interaction with UBC9 [30]. Surprisingly, we failed to detect an interaction between NHR-25 and CeUBC-9 either by Y2H assays or through immunoprecipitation of purified proteins (Figure S3B; data not shown). However, when we performed a yeast three-hybrid assay, where untagged CeSMO-1 was added to the system, we observed a weak interaction between NHR-25 and CeUBC-9, suggesting either that CeSMO-1 bridges NHR-25 and CeUBC-9 or that NHR-25 recognizes a CeSMO-1-bound CeUBC-9 species (Figure S3B).

### NHR-25 binds consensus sequences derived from NR5 family binding sites

**Figure 5:**
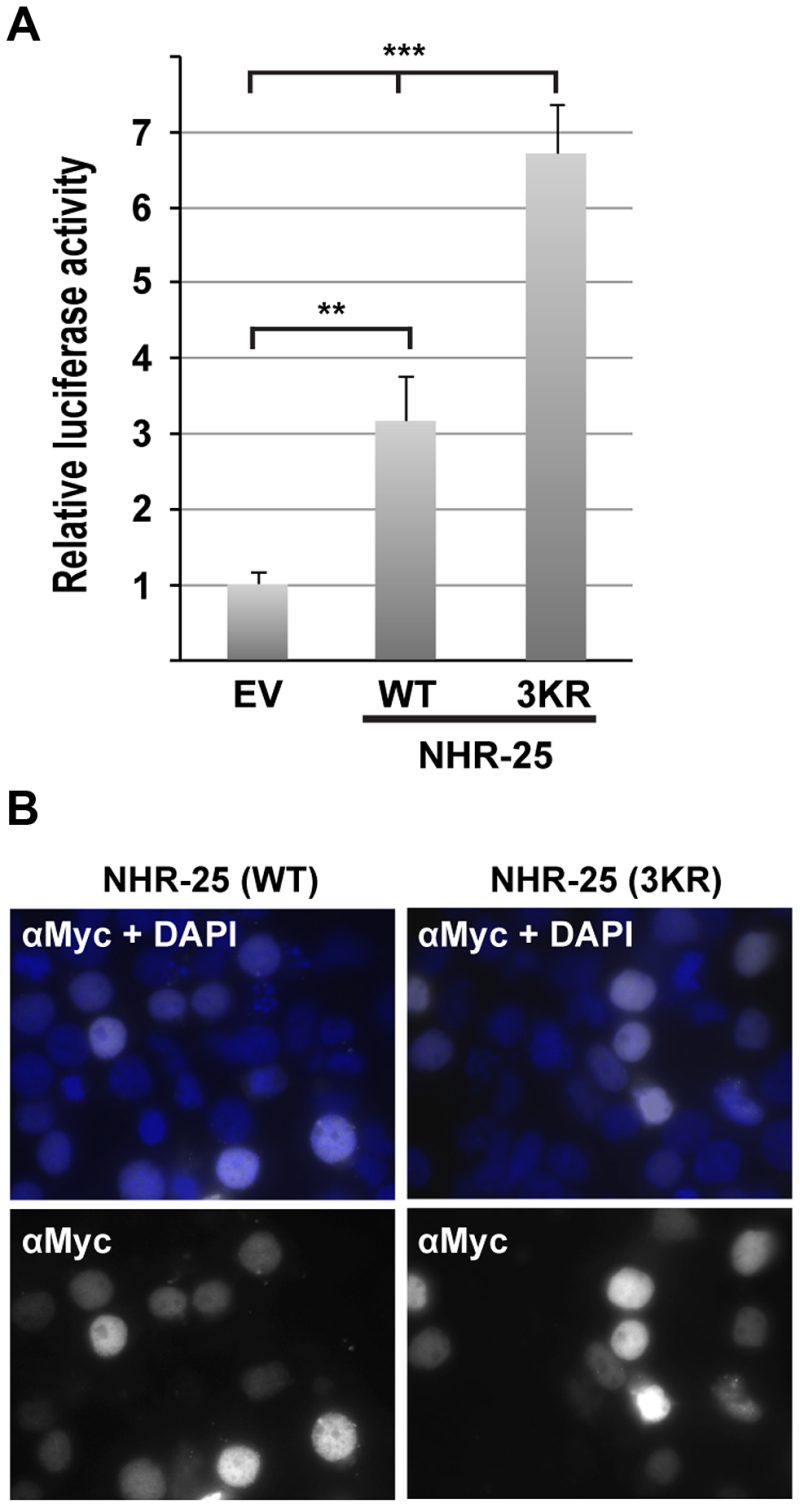
NHR-25(3KR) displays elevated activity in heterologous reporter assays. (A) A luciferase reporter vector containing four Ftz-F1/NHR-25 binding sites was transfected into HEK293T cells along with a Renilla internal control and either Myc-NHR-25 (WT) or Myc-NHR-25(3KR) expression constructs. Relative luciferase activity was normalized to the internal Renilla control and empty expression vector (EV). Eight biological replicates from three independent experiments were analyzed and error bars indicate standard deviation. (**T-test *p<0.01*; *** *p<0.0001*) (B) Transfected cells were stained with anti-Myc antibody. NHR-25(3KR) does not affect NHR-25 levels or localization. Nuclei were visualized with DAPI staining and an overlay of Myc and DAPI staining is shown.

To begin to investigate how sumoylation affects NHR-25-dependent transcriptional activity, we employed a HEK293T cell-based assay. We used a luciferase reporter driven by four tandem Ftz-F1 (*Drosophila* homolog of NHR-25) consensus sites, previously shown to be responsive to NHR-25 [8]. When Myc-tagged wild type NHR-25 was transfected, reporter expression was enhanced (Figure 5A), and the sumoylation-defective mutant NHR-25 (3KR) activated the reporter more strongly (Figure 5A). Anti-Myc immunostaining indicated no detectable increase in protein level or nuclear localization (Figure 5B).

To better characterize NHR-25-dependent transcriptional activity and generate reporters that could subsequently be used for *in vivo* assays, we generated a construct based on the canonical, high affinity SF-1 regulatory elements derived from the Mullerian inhibiting substance (MIS) and CYP11A1 (CYP) genes. We assessed NHR-25 binding to these elements using yeast one-hybrid (Y1H) and electrophoretic mobility shift assays (EMSAs). The Y1H assays indicated that NHR-25 bound the MIS and CYP11A1 elements (Figure S7A, B). Mutations in the MIS binding site that block SF-1 binding (MIS MUT) [27] prevented NHR-25 binding (Figure S7B). Moreover, the NHR-25 L32F (*ku217*) mutant, which has impaired DNA binding *in vitro* [7], displayed reduced activity in the Y1H experiment (Figure S7B). Consistent with the Y1H data, we found that a 6×His-MBP tagged fragment of NHR-25 (amino acids 1–173) purified from *E. coli* clearly bound MIS and CYP11A1 sites singly (Figure S7C) or in combination (2×NR5RE WT, for nuclear receptor NR5 family Response Element; Figure S7D) but only weakly to the mutant sites (Figure S7C–D, 7A).

**Figure 6:**
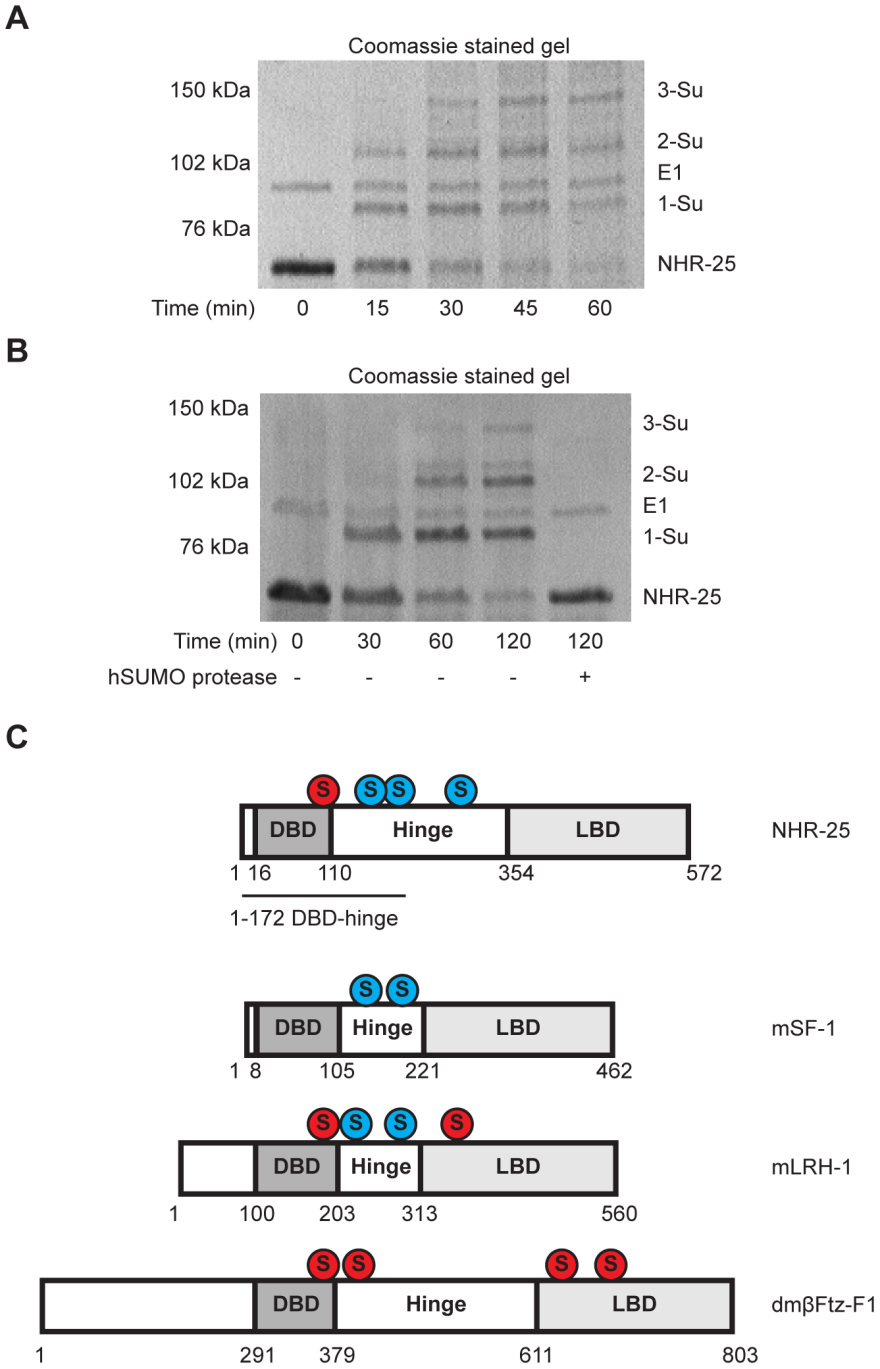
The NHR-25 DBD is robustly sumoylated. (A and B) *In vitro* sumoylation reactions were resolved by SDS-PAGE and Coomassie stained. A 6×His-MBP-NHR-25 (amino acids 1–173) substrate was used and incubated with hE1, hE2, and either CeSMO-1 (A) or methyl-hSUMO1 (B) for the indicated time in minutes. Methyl-hSUMO1 is a modified protein that blocks SUMO chain formation. Recombinant hSENP1 SUMO protease was included in (B) to demonstrate that bands reflected sumoylated species. A size standard in kilodaltons (kDa) is provided. (C) Schematic of sumoylation sites within NR5 family proteins. DNA-binding domains (DBD), hinge, and ligand-binding domains (LBD) are indicated. Sumoylation sites based on SUMOplot prediction and conservation in multi-species alignments are shaded red. SUMO acceptor lysines confirmed by *in vitro* biochemistry or cell-based sumoylation assays are shaded blue. The DBD-hinge fragment used in (A and B) is underlined.

Sumoylation of SF-1 regulates binding to specific DNA sequences [27]. Therefore, we asked whether sumoylation could similarly affect DNA binding capacity of the 6×His-MBP tagged fragment of NHR-25. We found that this fragment, which encompasses the DBD and part of the hinge region of NHR-25 (amino acids 1–173), was an even more potent sumoylation substrate than the hinge-LBD fragment, as almost all of the DBD substrate could be sumoylated (Figure 6A). Unlike SF-1 [27], NHR-25 DNA binding did not inhibit sumoylation (data not shown). Use of methyl-hSUMO1 in our *in vitro* sumoylation assays indicated that there were three sumoylation sites within the 6×His-MBP tagged fragment of NHR-25 DBD substrate (Figure 6B). These corresponded to the hinge region K165 and K170 acceptor lysines, which are analogous to the SF-1 fragment used by Campbell *et al.* (2008), and a third SUMO acceptor lysine (K84) within the DBD region between the second zinc finger and the conserved Ftz-F1 box (Figure 6C). This acceptor lysine is conserved in *D. melanogaster* Ftz-F1 as well as the mammalian LRH-1 (Figure 6C) [31]. EMSAs indicated that sumoylation diminished binding of the NHR-25 DBD fragment to the MIS and CYP derived binding sites (Figure S7D). Modifying the EMSAs such that the sumoylation reaction preceded incubation with the 2×NR5RE oligos severely impaired binding (Figure S7E). These *in vitro* findings are consistent with the notion that, as in mammals, sumoylation could diminish NHR-25 DNA binding.

### Sumoylation inhibits NHR-25 dependent transcription *in vivo*

**Figure 7:**
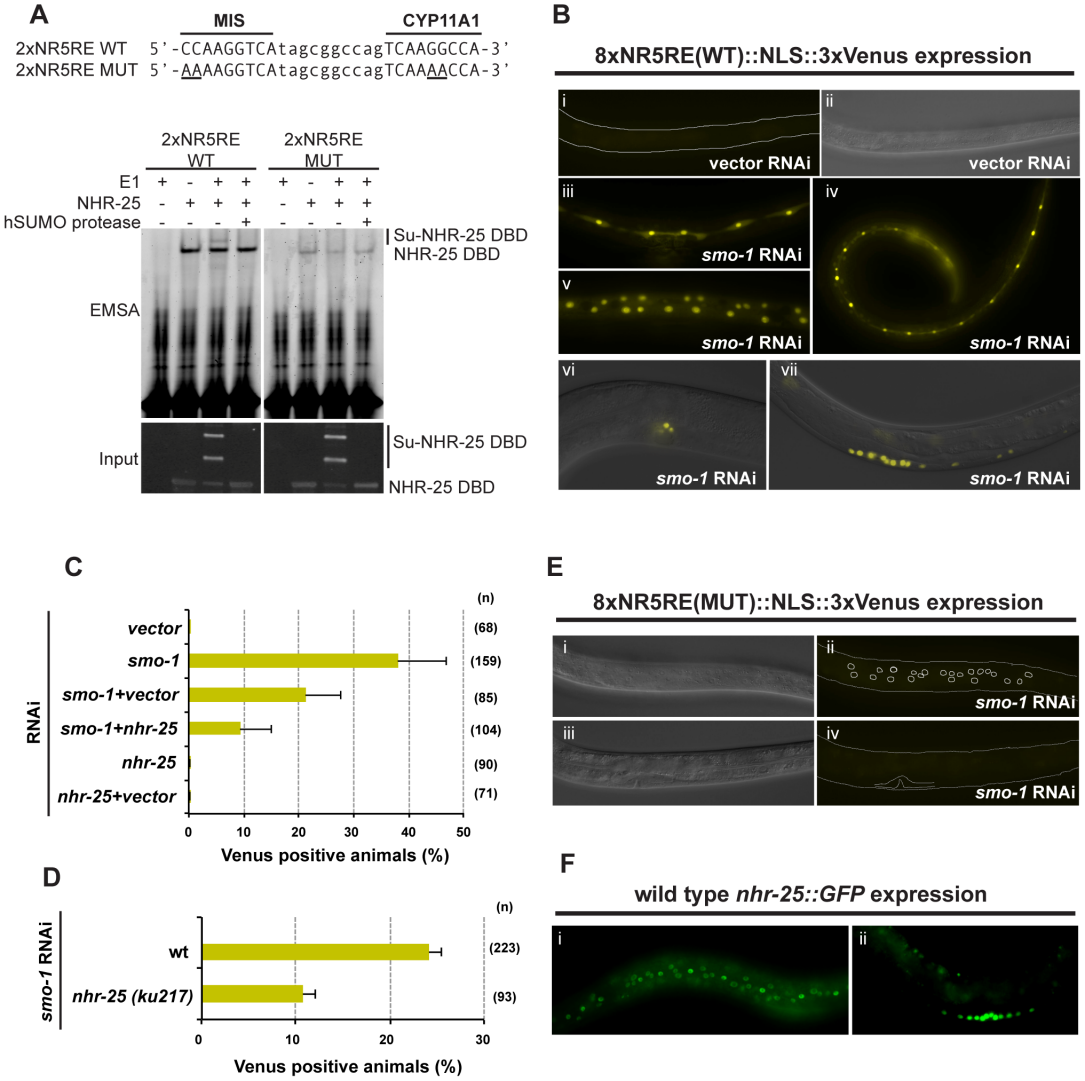
Sumoylation inhibits NHR-25-dependent transcription *in vivo*. (A) NHR-25 binds to canonical SF-1 target sequences. Sequence of the wild-type (WT) and mutant (MUT) MIS and CYP11A1 binding sites used are shown on top. Bases altered in the MUT sequences are underlined. The annealed 2×NR5RE oligonucleotides were incubated with combinations of the following: sumoylation enzymes (hUbc9+CeSMO-1) with or without hE1 enzyme, and NHR-25 DBD substrate. Recombinant hSENP1 SUMO protease was included to demonstrate that bands reflected sumoylated species. The corresponding proteins in the EMSA were detected by anti-MBP immunoblotting (input). The positions of unsumoylated and sumoylated NHR-25 DBD are indicated. (B) Animals carrying an *8×NR5RE (WT)::NLS::3×Venus* transgene as an extrachromosomal array were generated. No Venus expression was detected in transgenic animals on vector RNAi (i and ii). The nematode body is outlined in (i), and the corresponding differential interference contrast (DIC) image of the same animal is provided (ii). Representative Venus expression in transgenic animals treated with *smo-1* RNAi (iii–vii). Expression was observed in seam cells at L4 (iii), in seam cells and hyp7 at L3 (iv), in hyp7 at L4 (v), in the AC and vulF at early L4 (vi), and in developing vulval cells at L3 (vii). Fluorescent and DIC images were merged in vi and vii. (C and D) Transgenic animals expressing the Venus reporter in at least one of the following tissues: seam cells, hyp7, or vulval cells; were scored. Reduction of *nhr-25* function either by RNAi (C) or by *ku217* mutation (D) reduced the *8×NR5RE (WT)* reporter activity following *smo-1* RNAi. (n) number of animals scored. (E) Mutations (MUT) in NR5RE completely eliminated Venus expression following *smo-1* RNAi. DIC (i and iii) images corresponding to Venus fluorescence images (ii and iv, respectively) are provided. Positions of hypodermal nuclei (ii) and the developing vulva (iv) are outlined. (F) NHR-25::GFP is expressed in nuclei of seam cells and hyp7 (i) and the developing vulva (ii), similar to 8×NR5RE (WT)::NLS::3×Venus reporter expression. All animals are positioned with the anterior to the left.

We next wanted to assess the effects of sumoylation on NHR-25-dependent transcription *in vivo*. To enhance the sensitivity of our assays, we constructed a reporter carrying four tandem repeats derived from each of MIS and CYP genes (Figure 7A, eight SF-1/NHR-25 binding sites designated as 8×NR5RE). The binding sites were spaced ten base-pairs apart to facilitate potential cooperative binding [32]. We generated transgenic *C. elegans* carrying the 8×NR5RE positioned upstream of a *pes-10* minimal promoter and driving a 3×Venus fluorophore bearing an N-terminal nuclear localization signal. In wild type animals, reporter expression was not detected (Figure 7B), whereas after *smo-1* RNAi, strong expression was detected in developing vulval cells, the hypodermis, seam cells, the anchor cell (Figure 7B) and embryos (not shown), tissues in which NHR-25 is known to be expressed (Figures 7F) and functional [4], [7], [33]. Reporter expression was especially prominent during the L3 and L4 stages. Mutation of the binding consensus, 8×NR5RE(MUT) abolished reporter expression in a *smo-1* (RNAi) background (Figure 7E), as expected for NHR-25-dependent reporter expression. Moreover, genetic inactivation of *nhr-25* either by RNAi (*smo-1*, *nhr-25* double RNAi) or by use of *nhr-25(ku217)*, a reduction-of-function allele of *nhr-25*, abrogated reporter expression even in *smo-1* knockdown animals (Figure 7C, D). We conclude that sumoylation of NHR-25 strongly reduces its transcriptional activity *in vivo*.

### Sumoylation of NHR-25 prevents ectopic vulval development

To examine functionally the consequences of NHR-25 sumoylation, we returned to the roles of *nhr-25* and *smo-1* in vulval organogenesis. Noting that *smo-1* mutants but not *nhr-25* reduction-of-function mutants display a Muv phenotype, we investigated whether this might reflect enhanced NHR-25 activity due to its reduced sumoylation. We therefore generated transgenic animals expressing tissue-specific NHR-25 and/or SMO-1 driven by three different promoters; *egl-17* for the VPCs, *grl-21* for the hypodermal hyp7 syncytium, and *wrt-2* for the seam cells. These transgenes included (i) wild type NHR-25; (ii) NHR-25 3KR; or (iii) SMO-1 alone. Although *egl-17* is typically used as a 1° and 2° cell fate marker during vulva development, it is expressed in all VPCs in earlier stages [34](Figure S2C). We used the *egl-17* promoter rather than commonly used VPC driver, *lin-31*, because the heterodimeric partner of LIN-31 is sumoylated and directly involved in vulva development [28].

**Figure 8:**
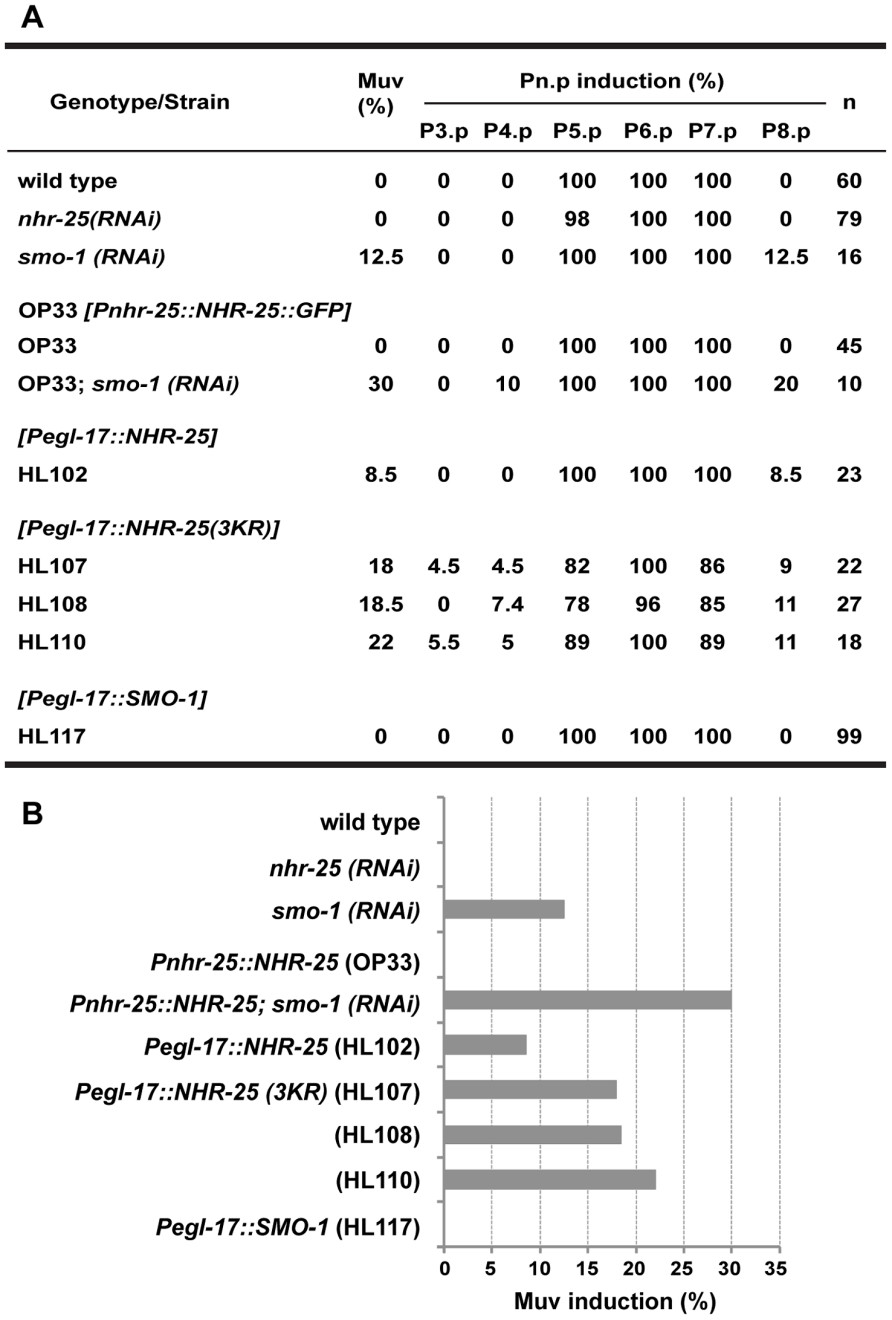
Overexpression of unsumoylated NHR-25 causes multivulva induction. (A) Table providing scoring of overall multivulva (Muv) induction in the indicated strains/genotypes, as well as induction in individual VPCs. Number of animals (n) scored for each strain genotype is provided. Use of brackets denotes transgenic genotypes. (B) Graphical representation of the overall percentage of animals for each strain that display Muv induction of any VPC.

Muv induction was scored by observing cell divisions of the six VPCs with the potential to respond to the LIN-3/EGF signal, which promotes differentiation. Normally, only P5.p, P6.p, and P7.p are induced while P3.p, P4.p and P8.p each produce no more than two cells as they are destined to fuse with the surrounding hyp7 syncytium (Figure 2). In wild type animals, overexpression of NHR-25 in the VPCs (*egl-17* promoter) but not in hyp7 or seam cells (*grl-21* and *wrt-2* promoters, respectively) drove Muv induction at the P8.p position, mimicking *smo-1* RNAi (Figure 8, Table S1). Thus, high level NHR-25 acted cell-autonomously to produce a Muv phenotype. Overexpression of the NHR-25 3KR mutant in the VPCs resulted in an even more penetrant Muv phenotype and greater induction of P3.p, P4.p, and P8.p (Figure 8A). In contrast, overexpression of SMO-1 alone did not produce the Muv phenotype.

These overexpression experiments implied that excess unsumoylated NHR-25 altered 3° VPC fate, permitting extra divisions that produce the Muv phenotype. If sumoylation of NHR-25 normally constrains its activity, animals with decreased sumoylation activity would be expected to enhance the Muv phenotype. To test this hypothesis, we assessed the effect of *smo-1* RNAi in animals expressing a low-copy, integrated transgene expressing C-terminally GFP-tagged NHR-25 [35]. This transgene likely recapitulates the expression pattern of endogenous *nhr*-25, since the construct includes the complete 20 kb intergenic region upstream of *nhr-25*, and the entire *nhr-25* gene and 3′-UTR; the animals display normal vulvas. However, exposure to *smo-1* RNAi caused the Muv phenotype in about 30% of animals carrying the *nhr-25::gfp* transgene, which exceeded the 12% Muv frequency in *smo-1* RNAi controls (Figure 8). This extra vulva induction was seen in the P4.p. lineage in addition to P8.p. Together, our findings strongly suggest that in wild type animals, NHR-25 sumoylation prevents ectopic vulva induction in 3° fated cells.

### Effects of *smo-1* deficiency on NHR-25 expression

**Figure 9:**
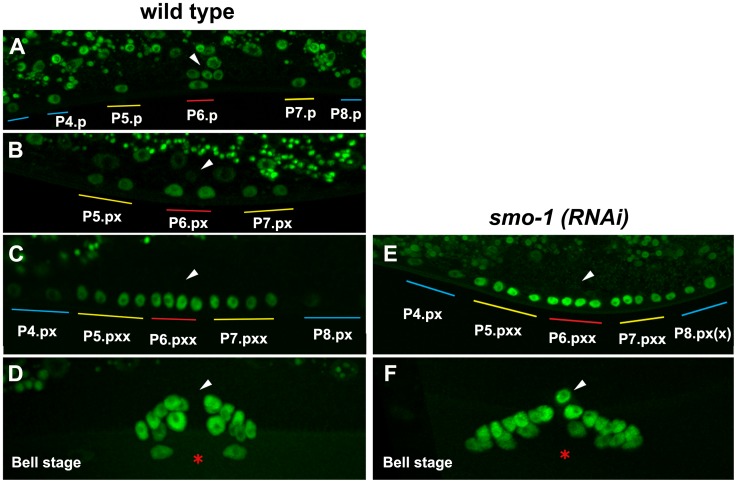
NHR-25::GFP (OP33) expression during vulval development. Expression in 1-cell stage Pn.p cells (A), in 2-cell stage Pn.px cells (B) and 4-cell stage Pn.pxx cells (C) in wild type and in *smo-1(RNAi)* animals (E). Higher levels and ectopic expression of NHR-25 were seen in P4.px and P8.px(x) in a *smo-1(RNAi)* background (E). Expression at the bell stage in wild type and *smo-1(RNAi)* animals (D,F). Ectopic expression in the AC observed in *smo-1(RNAi)* animals. Arrowheads indicate the position of the AC, red asterisk indicates the position of the invaginated vulva. Colored bars indicate 1° (red), 2° (yellow), and 3° (blue) lineages, as described in Figure 2.

One interpretation of our genetic and biochemical data is that the *in vivo* ratio of sumoylated to non-sumoylated NHR-25 specifies or maintains the 3° VPC fate. We were therefore interested in how NHR-25 sumoylation was regulated. SMO-1 is expressed at constant levels throughout vulval development [19], so we examined whether NHR-25 levels were regulated in VPCs during development. The low-copy, integrated NHR-25::GFP translational fusion allowed us to examine the developmental pattern of NHR-25 expression. NHR-25::GFP was evenly distributed prior to the first division in all VPCs, whereas after the first division the pattern became graded: highest in 1° P6.p daughters, lower in 2° P5.p and P7.p daughters, and lowest in 3° P(3,4,8).px (Figure 9A, B). After the third round of cell divisions NHR-25::GFP expression continued in all 22 P(5–7).pxxx cells and remained high during early vulva morphogenesis (Figure 9D) until it temporarily disappeared by the “Christmas tree stage” (data not shown).

*smo-1* RNAi caused ectopic NHR-25::GFP expression in P(4,8).pxx cells (Figure 9E), which displayed the strongest Muv induction in *NHR-25::GFP;smo-1(RNAi)*, and *Pegl-17::NHR-25(3KR)* backgrounds (Figure 8). In wild type animals, NHR-25::GFP was normally expressed in the anchor cell at the time of the first VPC divisions, and subsequently decreased (Figure 9D). Interestingly, we noted that in nine of ten *smo-1(RNAi)* animals NHR-25::GFP was re-expressed in the AC at the “bell stage” (Figure 9F). Subsequently, no AC invasion occurred and the AC remained unfused. Therefore, in addition to restricting NHR-25 activity in 3° cells (previous section), sumoylation also limits NHR-25 accumulation in cells that are destined to assume the 3° fate. The resultant NHR-25 gradient combined with constant levels of SMO-1 may account for the observed pattern of NHR-25 sumoylation.

## Discussion

The capacity of TFs to specify expression of precise networks of genes in a given context, yet remain flexible to govern dramatically different sets of genes in different cell or physiologic contexts, likely involves combinatorial regulation of transcription. In this study, we show that sumoylation represses bulk NHR-25 activity in multiple *C. elegans* tissues. In addition, our findings suggest that particular fractional sumoylation states of NHR-25 govern the appropriate course of cell divisions and the 3° fate decision of vulval precursor cells, thereby determining morphogenesis of the entire organ.

### Balance of NHR-25 sumoylation in vulval morphogenesis

Supporting the notion that sumoylation can constrain NHR-25 activity, we found that a reporter fusion responsive to NHR-25 was strongly upregulated upon depletion of *smo-1* by RNAi (Figure 7B). Our *in vitro* findings suggested that sumoylation of NHR-25 diminished DNA binding (Figure S7), while our *in vivo* studies suggested that reduction of *smo-1* caused ectopic accumulation of NHR-25 (either synthesis or impaired degradation) in VPCs P4.p and P8.p (Figure 9). These data suggest two modes, not mutually exclusive, through which sumoylation can regulate NHR-25. Moreover, overexpression of either NHR-25 or its sumoylation-defective form (NHR-25 3KR) led to multivulva induction in cells that normally adopt the 3° fate (Figure 8).

**Figure 10:**
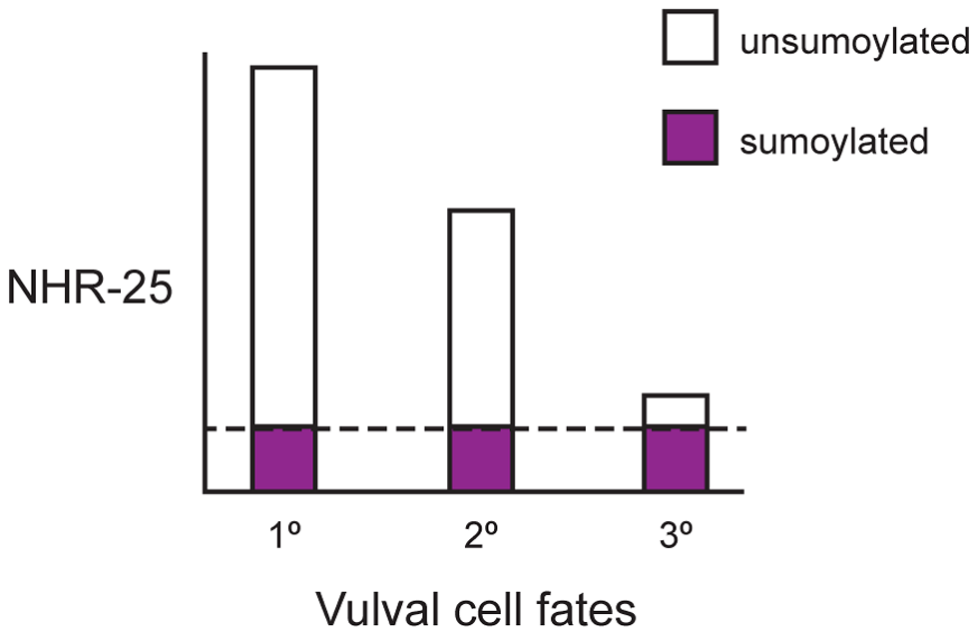
Ratio of sumoylated to unsumoylated NHR-25 and 3° cell fate. After the first round of cell division, VPCs adopt 1°, 2°, and 3° fates and NHR-25 accumulates in a gradient. The highest NHR-25 levels are in 1° fated cells, lower NHR-25 levels are in 2° fated cells, and the lowest levels are in 3° fated cells. Sumoylation output is a reflection of the combined activities of the sumoylation machinery and the SUMO proteases. In this model, sumoylation output is limiting, and the NHR-25 gradient results in a gradient of unsumoylated NHR-25. 1° cells have the highest ratio of unsumoylated to sumoylated NHR-25, and the ratio decreases as NHR-25 levels drop in 2° and 3° VPCs. The dashed line indicates the constant amount of sumoylated NHR-25 produced by limiting, steady-state sumoylation. At a particular threshold, enough sumoylated NHR-25 relative to unsumoylated NHR-25 allows 3° cells to either adopt and/or maintain the correct fate.

Together, our data support a model in which proper differentiation of VPCs depends on the appropriate balance of sumoylated and unsumoylated NHR-25 (Figure 10). Importantly, NHR-25 affects VPC specification cell-autonomously, as overexpression of NHR-25 in other epidermal cells, such as the seam cells or hyp7, did not cause a Muv phenotype (Table S1). Furthermore, NHR-25 appears to form a gradient across the VPC array, accumulating to high levels in 1° fated cells, intermediate levels in 2° fated cells and low levels in 3° fated cells (Figure 9). Our findings indicate that sumoylation promotes a specific pattern of NHR-25 activity in differentially fated VPCs and the relative level of NHR-25 sumoylation is critical for promotion and/or maintenance of the 3° cell fate (Figure 10).

The role(s) of NHR-25 and SMO-1 in vulval induction are likely pleiotropic. Multiple vulval development factors are sumoylated [22], [28], [29], [36], including LIN-11, which is responsible in part for promoting vulval-uterine fusion [19]. Based on expression pattern and phenotypes, NHR-25 likely acts in other cell-types (hyp7, 1°/2° VPCs, or AC) and at different developmental time points to regulate vulval induction. The Muv phenotype of *smo-1*-deficient animals was enhanced by *nhr-25* RNAi (Figure 1). Synthetic multivulva (synMuv) genes inhibit *lin-3* activity in the syncytial hyp7 cell to prevent aberrant vulva induction in the neighboring 3° cells [37]. Yet, overexpression of NHR-25 in the hyp7 syncytium did not cause Muv induction (Table S1), thus it is unlikely that NHR-25 acts through this pathway. Our overexpression data indicates that NHR-25 acts cell-autonomously in the VPCs (Figure 8), and likely interacts with canonical signaling pathways that promote VPC fate. The NHR-25 expression gradient is reminiscent of the LIN-3/EGF gradient which promotes vulval induction through Ras activation and subsequent Notch signaling [38]. *nhr-25* appears to act downstream of LET-60/Ras signaling, as gain-of-function LET-60/Ras causes elevated NHR-25 expression (data not shown). However, regulation of *lin-3* by NHR-25 in the anchor cell has also been suggested [39]. Ectopic expression of NHR-25 in the AC following *smo-1* RNAi is unlikely to cause Muv induction since, developmentally, this expression occurs much later than VPC fate determination. In wild type animals, NHR-25 levels are therefore downregulated in the AC, which may be required for proper completion of AC invasion and/or fusion. Additionally, the cell division arrest seen in *nhr-25* RNAi leading to the Pvl phenotype was enhanced by inactivation of *smo-1* (Figure 1). For instance, the Pvl phenotype can arise from *nhr-25* reduction of function, which causes defective 1° and 2° cell divisions (Figure 1, Table 1), or from *smo-1(lf)*, which impairs uterine-vulval connections [19]. Thus, an exquisite interplay between various sumoylated targets as well as the balance between sumoylated and unsumoylated NHR-25 collaborate to ensure proper vulval formation.

How could unsumo∶sumo NHR-25 balance regulate 3° cell fate? Sumoylation might alter NHR-25 levels or activity in a manner that shifts the unsumo∶sumo NHR-25 ratio, which in turn acts as a switch to determine NHR-25 output. The activities of a mammalian nuclear hormone receptor have been shown to shift dramatically with signal-driven changes in levels of receptor activity [40]. Another possibility is that the sumoylated and unsumoylated versions of NHR-25 regulate distinct targets, and the unsumo∶sumo ratio in different cells thereby determines the network of NHR-25-regulated genes. Indeed, sumoylation appears to affect the genomic occupancy of the NHR-25 ortholog SF-1 [27]. We note that NHR-25 sumoylation could be context-dependent. Sumoylation could increase NHR-25 activity at particular response elements. Accordingly, sumoylation positive regulates the activity of the nuclear hormone receptors RORα and ER [41], [42].

The finding that overexpression of NHR-25 strongly provoked a Muv phenotype suggests that sumoylation state of NHR-25 in VPCs is exquisitely regulated. Such regulation might be accomplished by subtle changes in availability of SUMO in different VPCs, not detected by our assays, or by the relative activities of the sumoylation machinery and the SUMO proteases. A similar competition for constant levels of SUMO regulates Epstein-Barr virus infections, where the viral BZLF protein competes with the host PML protein for limiting amounts of SUMO1 [43].

### Sumoylation as a nuclear hormone receptor signal

It is intriguing to consider SMO-1 as an NHR-25 ligand parallel to hormones or metabolites bound noncovalently nuclear hormone receptors in other metazoans, and by the *C. elegans* DAF-12 receptor. Indeed, such expansion of the concept of signaling ligands could “de-orphan” many or all of the 283 *C. elegans* nuclear hormone receptors for which no traditional ligands have been identified. Detection of noncovalent ligands is very challenging; numerous mammalian NHRs remain “orphans” despite intensive efforts to find candidate ligands and evidence that the ancestral NHR was liganded [44]. In principle, SUMO can be conjugated to its target sequence motif anywhere on the surface of any protein, whereas classic NHR ligands bind only stereotyped pockets within cognate NHR LBDs. Viewed in this way, SUMO may directly regulate many NHRs (and other factors as well), whereas classical NHR ligands act more selectively on only one or a few NHRs. The multifactorial regulation of NHRs would provide ample opportunity for gene-, cell- or temporal-specificity to be established in cooperation with the SUMO ligand.

### Modes of SUMO regulation in *C. elegans*

There are three ways in which SUMO can potentially interact with target proteins: i) non-covalent binding, where a protein binds either free SUMO or SUMO conjugated onto another protein; ii) sumoylation, where SUMO associates covalently with a target protein through an isopeptide linkage; and iii) poly-sumoylation, where chains of SUMO are built up from an initially monosumoylated substrate. In *C. elegans*, SMO-1 can bind proteins non-covalently [45] or can be covalently linked to substrates (Figure 4). Polysumoylation occurs through SUMO modification of acceptor lysines within SUMO proteins [46]. In our assays, we saw no robust polyCeSMO-1 chains compared to the hSUMO2 control, even after prolonged reaction times (Figure S6). Consistent with this result, sumoylation motifs were predicted within hSUMO1, 2 and 3, and yeast SMT3 but not in CeSMO-1. PolySUMO chains in yeast and vertebrates can be recognized by SUMO targeted ubiquitin ligases (STUbLs) that polyubquitinate the polySUMO chain and direct it for degradation by the 26S proteasome [46]. Judging from BLAST analysis, there are no evident homologs of the known STUbLs hsRNF4 or yeast SLX5–8 in *C. elegans*. As both *S. cerevisiae* SUMO (SMT3) and vertebrate SUMO2 and SUMO3 form polySUMO chains, it appears that *C. elegans* has lost the ability to form polySUMO chains.

### Functional homology with SF-1/LRH-1

The mammalian homologs of NHR-25 (SF-1 and LRH-1) are sumoylated on two sites within the hinge region of the protein, between the DBD and LBD [21], [27], [47]. These SUMO acceptor sites occur at corresponding positions in NHR-25, with the site near the DBD being duplicated (Figure 6C). Additionally, our DBD sumoylation experiments suggest the presence of a fourth sumoylation site in NHR-25, conserved with *D. melanogaster* Ftz-F1 and mammalian LRH-1 (Figure 6C) [7], [31]. Thus, NHR-25 appears to have sumoylation sites that are conserved in both SF-1 and LRH-1 as well as at least one site that is only conserved in LRH-1. Similarly, NHR-25 seems to combine regulation of processes that in mammals are either regulated by SF-1 only or LRH-1 only. Additionally, human SUMO1 can be conjugated onto NHR-25 and *C. elegans* SMO-1 can be conjugated onto SF-1 (Figure 4, S4). Therefore, despite the 600–1200 million years of divergence since the common ancestor of humans and nematodes, regulation of NR5A family by sumoylation appears to be incredibly ancient. There are also, however, notable differences. For instance, while LRH-1 and SF-1 strongly interact with UBC9, providing a mechanism for robust, E3 ligase-independent sumoylation [20], this did not appear to be the case for NHR-25. As indicated above, we also did not find evidence for polysumoylation of NHR-25.

Having established SUMO as an NHR-25 signal that regulates cell fate, it will be exciting to further explore how sumoylation affects the NHR-25 gene regulatory network. It will be essential in future work to identify direct NHR-25 target genes by ChIP-seq, to determine how sumoylation impacts NHR-25 response element occupancy, and to mutate sumoylation sites and response elements with genome editing technologies, such as CRISPR/Cas9 [48]. The compact *C. elegans* genome facilitates unambiguous assignment of putative response elements to regulated genes, a daunting challenge in vertebrate systems. Further, the extensive gene expression and phenotypic data accessible to the *C. elegans* community will allow identification of candidate NHR-25 target genes directly responsible for regulating animal development and physiology. Understanding how NHR-25 sumoylation regulates specific genes, and how this information is integrated into developmental circuits will advance our understanding of combinatorial regulation in metazoan gene regulatory networks.

## Materials and Methods

### Molecular biology

cDNAs and promoters/binding sites were Gateway cloned (Invitrogen) into pDONR221 and pDONR-P4P1r, respectively. Mutations were introduced into the *nhr-25* cDNA using site-directed mutagenesis with oligonucleotides carrying the mutation of interest and Phusion polymerase (NEB). cDNAs and promoters were then moved by Gateway cloning into destination vectors. NHR-25 (amino acids 161–541) and NHR-25 (amino acids 1–173) were moved into the bacterial expression vector pETG-41A, which contains an N-terminal 6×His-MBP tag. CeUBC-9 and CeSMO-1 cDNAs were moved into the bacterial expression vector pETG-10A, which contains an N-terminal 6×His tag. The CeUBC-9 construct also carried an N-terminal tobacco etch virus (TEV) cleavage site for removal of the 6×His tag, similar to the hUBC9 bacterial expression construct. For Y1H experiments, 2×SF-1 binding sites were Gateway cloned into pMW2 and pMW3 [49]. For Y2H experiments, cDNAs were moved into pAD-dest and pDB-dest [18], which contain the Gal4 activation domain and DNA binding domain, respectively. For Y3H, *smo-1* was moved into pAG416-GPD-ccdB-HA [50], which results in constitutive expression. For luciferase experiments, cDNAs were moved into pDEST-CMV-Myc. For our *C. elegans* expression experiments, cDNA constructs were Gateway cloned into pKA921 along with either the *egl-17*, *wrt-2*, or *grl-21* promoter. The *egl-17* promoter was PCR cloned from N2 genomic DNA. The *wrt-2* and *grl-21* promoters (pKA279 and pKA416, respectively) were previously cloned [12]. pKA921 contains a polycistronic mCherry cassette to allow monitoring of construct expression. For our 3×Venus reporters, three-fragment Gateway cloning into pCFJ150 [51] was performed. The *8×NR5RE-pes-10Δ* promoter fragments were cloned into pDONR-P4P1r. *C. elegans* codon optimized *3×Venus* was cloned from *Prnr::CYB-1DesBox::3×Venus* [52] and an NLS was added on the 5′ end of the gene and *NLS-3×Venus* was Gateway cloned into pDONR221. The *unc-54* 3′-UTR in pDONR-P2rP3 was a gift from the Lehner lab. Primer sequences are provided in Table S2. Plasmids generated for this study are listed in Table S3.

### Y2H screening and matrix assays and Y1H analyses

Yeast transformations and Y2H assays were carried out as described by Deplancke *et al.* [53]. For the Y2H screen, *S. cerevisiae* strain MaV103 carrying a pDB-*nhr-25* construct was transformed with 100 ng of the AD-Orfeome cDNA library, in which 58% of the known *C. elegans* open reading frames are fused to the Gal4 activation domain [18]. Six transformations were performed per screen and 149,800 interactions were screened, representing 12.5-fold coverage of the library. Positive interactions were selected for by growth on SC dropout plates lacking leucine, tryptophan, and histidine; these plates were supplemented with 20 mM of the histidine analog 3-aminotriazole. Interactions were confirmed by β-galactosidase staining. We identified 42 candidate interactors, but only *smo-1* was recovered multiple times (seven independent isolations). Moreover, upon cloning and retesting the candidate interactor cDNAs, only *smo-1* was confirmed as an interactor. The screen identified no other components of the SUMO machinery or known SUMO binding proteins. Generation of Y1H bait strains and Y1H analyses were performed as described [53]. pDB constructs carrying NHR-2, NHR-10, NHR-31, NHR-91, NHR-105, FAX-1, and ODR-1 cDNAs were a gift from Marian Walhout.

### Protein purification

Recombinant hE1, hUBC9, hSUMO1, hSUMO2, hSENP1, and murine SF-1 LBD were purified as described [27], [54]–[56]. 6×His-CeSMO-1 and 6×His-TEV-CeUBC-9 were expressed in BL21(λDE3) *E. coli* and purified using a similar scheme as used to purify their human counterparts [55], [56]. 6×His-MBP-NHR-25 (amino acids 161–541) was freshly transformed into BL21(λDE3) *E. coli*. A 1 L culture was grown to an OD600 of ∼0.8, induced with 0.2 mM isopropylthio-β-galactoside (IPTG), and shaken at 16°C for four hours. Bacteria were lysed using a microfluidizer in 20 mM Tris-HCl pH 8.0, 350 mM NaCl, 20 mM imidazole containing EDTA-free Protease Inhibitor Cocktail III (EMD Millipore). 6×His-MBP-NHR-25 was then purified using nickel affinity chromatography (5 ml His Trap FF column, GE Healthcare). Peak fractions were pooled, dialyzed into 20 mM HEPES (pH 7.5), 1 mM EDTA, and 2 mM CHAPS {3-[(3-cholamidopropyl)-dimethylammonio]-1-propanesulfonate}, and purified by anion-exchange chromatography using a MonoQ column (GE Healthcare) and eluted with a 1 M ammonium acetate gradient. Peak fractions were pooled, concentrated and 6×His-MBP-NHR-25 was purified by size-exclusion chromatography using an S200 column (GE Healthcare). Peak fractions containing 6×His-MBP-NHR-25 were pooled, concentrated, dialyzed into 20 mM Tris pH 7.5, 50 mM NaCl, 10% glycerol, flash frozen in liquid nitrogen, and stored at −80°C. Later purifications used only nickel affinity chromatography. Using this preparation in sumoylation assays produced results similar to those obtained using the preparations purified over the three aforementioned columns. 6×His-MBP-NHR-25 (amino acids 1–173) was expressed and purified using a single nickel affinity chromatography step, as described above for the 6×His-MBP-NHR-25 (amino acids 161–541) fragment.

### *In vitro* sumoylation assays

Reactions were performed as described by Campbell *et al.* [27]. Briefly, 50 µl sumoylation reactions were set up with 0.1 µM E1, 10 µM UBC9, and 30 µM SUMO in a buffer containing 50 mM Tris-HCl (pH 8.0), 100 mM NaCl, 10 mM MgCl2, 10 mM ATP, and 2 mM DTT. Substrates were added at 1 µM and when required, 2.5 µg of hSENP1 SUMO protease was added. When *in vitro* transcribed proteins were used as substrates, 50 µl reactions were generated using a TnT T7 Quick Coupled Transcription/Translation System (Promega). 16 µl of this reaction was then used as a substrate in a 25 µl sumoylation reaction using the same molarities as described above. When SUMO protease was required, 1.25 µg of hSENP1 was added. Reactions were incubated at 37°C for the desired time, and stopped by boiling in protein sample buffer (10% Glycerol, 60 mM Tris/HCl pH 6.8, 2% SDS, 0.01% bromophenol blue, 1.25% beta-mercaptoethanol). Proteins were resolved by SDS-PAGE on either 4–12% Bis-Tris gradient gels (Invitrogen) or 3–8% Tris acetate gels (Invitrogen) followed by either Coomassie staining or immunoblotting. For immunoblotting, anti-NHR-25, anti-guinea pig-HRP (Santa Cruz), and anti-guinea pig-IR800 (Li-Cor) antibodies were used. Blots were developed using a LAS500 imager (GE Healthcare) or an Odyssey laser scanner (Li-Cor).

### Electrophoretic mobility shift assay (EMSAs)

Reactions were performed as described by Campbell *et al.* [27] with the following alterations. We added 400 µg/ml of bovine serum albumin to the EMSA buffer (50 mM Tris (pH 8.0), 150 mM NaCl, 10 mM MgCl_2_, 10 mM DTT, 10 mM ATP, and a 1 µM concentration of double-stranded oligonucleotide). Sequences of oligonucleotides are provided in Table S2. Oligonucleotides were annealed and then centrifuged in an Amicon Ultra 0.5 ml centrifugal filter (MWCO 50). Sumoylation reactions were set up on ice and added directly to the annealed oligonucleotides (20 µl final volume). Standard reactions used 500 nM of unmodified NHR-25 substrate, titration experiments added NHR-25 in 100 nM increments from 200–700 nM. At this point SENP1 (0.5 µl) was added when appropriate. We incubated these reactions at room temperature for 30 minutes to allow both sumoylation and DNA binding to occur. Half of the EMSA reaction (10 µl) was removed and added to 2 µl of 4× protein sample buffer and denatured by boiling for five minutes. Sumoylation products in the input were analyzed by immunoblotting using anti-MBP (NEB) and anti-mouse-IR800 (LiCor) antibodies. Blots were imaged using an Odyssey laser scanner. The remaining EMSA reaction was resolved on a 4–20% TBE polyacrylamide gel (Invitrogen) at 200 volts and stained with 1× SYBR Gold (Molecular Probes) in 0.5× TBE. Gels were then imaged using a Typhoon laser scanner (GE Healthcare).

### *C. elegans* culture and strains

*C. elegans* was cultured at 20°C according to standard protocols and the wild type strain is the N2 Bristol strain [57]. The following mutant and transgenic strains were used in this study: PS3972 *unc-119(ed4) syIs90 [egl-17::YFP+unc-119(+)]*, OP33 *unc119 (ed3); wgIs33 [nhr-25::TY1::EGFP::3×FLAG(92C12)+unc-119(+)]*, VC186 *smo-1(ok359)/szT1[lon-2(e678)]; +/szT1*, MH1955 *nhr-25(ku217)*. The following transgenic strains were generated for this study: HL102 *jmEx102[Pegl-17::Myc::NHR-25_mCherry+rol-6(su1006)]*, HL107, HL108, HL110 are independent lines carrying *jmEx107[Pegl-17::Myc::NHR-25(3KR)_mCherry+rol-6(su1006)]*, HL117 *jmEx118 [Pegl-17::Myc::SMO-1_mCherry+rol-6(su1006)]*, HL111 and HL112 are independent lines carrying *jmEx111[Pgrl-21::Myc::NHR-25_mCherry+rol-6(su1006)]*, HL121 *jmEx121[Pgrl-21::Myc::SMO-1_mCherry+rol-6(su1006)]*, HL113 and HL114 are independent lines carrying *jmEx113[Pwrt-2::Myc::NHR-25_mCherry+rol-6(su1006)]*, HL115 and HL116 are independent lines carrying *jmEx115[Pwrt-2::Myc::SMO-1_mCherry+rol-6(su1006)]*, HL153 *jmEx153[8×NR5RE (WT):pes-10Δ:NLS-3×Venus:unc-54 3′-UTR+Pmyo-2::tdTomato]*, HL155 *jmEx155[8×NR5RE (MUT):pes-10Δ:NLS-3×Venus::unc-54 3′-UTR+Pmyo-2::tdTomato]*, HL170 *nhr-25(ku217); jmEx153*.

### Constructs and microinjection

The following Gateway-based constructs were generated in pKA921: pJW522*[Pegl-17(1914 bp)::Myc::NHR-25_polycistronic_mCherry]*, pJW774 *[Pegl-17(1914 bp)::Myc:: NHR-25(3KR)_polycistronic_mCherry]*, pJW773 *[Pegl-17(1914 bp)::Myc::SMO-1_polycistronic_mCherry]*, pJW526 *[Pgrl-21(746 bp)::Myc::NHR-25_polycistronic_mCherry]*, pJW775 *[Pgrl-21(746 bp)::Myc::SMO-1_polycistronic_mCherry]*, pJW524*[Pwrt-2(1380 bp)::Myc::NHR-25_polycistronic_mCherry]*, pJW776*[Pwrt-2(1380 bp)::Myc::SMO-1_polycistronic_mCherry]*. The following Gateway-based constructs were generated in pCFJ150 [51]: pJW1109 *[8×NR5RE(WT):pes-10Δ:NLS-3×Venus:unc-54 3′-UTR]* and pJW1110 *[8×NR5RE(MUT):pes-10Δ:NLS-3×Venus::unc-54 3′-UTR]*. Plasmids were prepared using a PureYield Plasmid Midiprep System (Promega) followed by ethanol precipitation, or a Qiagen Plasmid Midi kit (Qiagen). Transgenic strains were generated by injecting 50 ng/µl of each plasmid into the *C. elegans* gonad [58] with the co-injection marker pRF4 [59]. For *8×NR5RE* reporter strain generation, N2 animals were injected with 30 ng/µl of the reporter plasmid and 5 ng/µl of co-injection marker *Pmyo-2::tdTomato* [60].

### RNA interference

Feeding RNAi was performed as described, with the indicated alterations to the protocol [61]. dsRNA was initially induced for four hours in liquid culture using 0.4 mM IPTG, before bacteria were concentrated and seeded on plates also containing 0.4 mM IPTG. Bacteria carrying pPD129.36 without an insert were used for control RNAi. For *nhr-25* RNAi, synchronized L2 larvae (19–20 hours after hatching) were fed on bacteria expressing *nhr-25* dsRNA to bypass the anchor cell (AC) defect. *smo-1* RNAi was performed on late L4 or young adults. For *in vivo* reporter assays, sodium hypochlorite-treated eggs were placed on RNAi plates seeded with dsRNA induced bacteria.

### Scoring VPC induction, lineaging and microscopy

To score vulva induction, nematodes were anesthetized in 10 mM levamisole, mounted onto 5% agar pads (Noble agar, Difco) and the number of daughter cells for each VPC were counted under differential interference contrast (DIC) optics. For lineaging analyses, the division pattern was followed under DIC from the two to eight cell stages [62]. Animals were mounted onto 5% agar pad with bacteria in S-basal medium without anesthesia. Olympus Fluoview FV1000 and Zeiss Axioplan microscopes were used for observation and imaging.

### NHR-25 antibody

A peptide-based anti-NHR-25 antibody was raised in guinea pig (Peptide Specialty Laboratories, GmbH, Germany). Animals were immunized against four short peptides in the hinge and LBD regions: PEHQVSSSTTDQNNQINYFDQTKC (24 a.a. 141–163); SLHDYPTYTSNTTNC (15 a.a. 250–263); TSSTTTGRMTEASSC (15 a.a. 283–296) RYLWNLHSNXPTNWEC (16 a.a. 507–521).

### Cell culture and luciferase assay

Human embryonic kidney (HEK) cell line 293T was maintained in Dulbecco's modified Eagle's medium (DMEM, Gibco), supplemented with 10% fetal bovine serum. Transfections were performed with polyethyleneimine (25 kDa, Sigma). The transcriptional activity of NHR-25 was tested with a luciferase vector carrying a CMV basic promoter driven by two copies of the Ftz-F1 binding consensus sequences TGAAGGTCA and TCAAGGTCA (total of four binding sites, 2×TGA-TCA::Luc) [8], [63]. Cells were seeded onto 24-well plates and the next day were transfected for three hours with a polyethylenimine mixture containing 50 ng of pTK-Renilla plasmid (Promega) as an internal control, 300 ng of the luciferase reporter plasmid, and 150 ng of the appropriate expression vector. The total amount of DNA was kept constant (1 µg) by adding empty expression vector where necessary. Forty hours post-transfection, the cells were harvested and processed using the Dual Luciferase Reporter Assay System (Promega). Eight independent biological replicates from three independent experiments were assayed, and data were presented as average values with standard deviations after normalization against the Renilla luciferase activities. For immunocytochemistry, transfected cells were fixed with 4% formaldehyde (Sigma) for 10 min. After washing with PBS, cells were permeabilized with PBS containing 0.2% TritonX-100 in (PBST), washed with TBST buffer (25 mM Tris-HCl, pH 7.5, 136 mM NaCl, 2.7 mM KCl and 0.1% TritonX-100), incubated in blocking solution (2.5% skim milk and 2.5% BSA in TBST). Anti-Myc 9E10 antibody (Sigma; 1∶2000 dilution) was added and incubated for overnight at 4°C. Following washing, goat-anti-mouse-TRITC conjugated 2° antibody (Jackson ImmunoResearch; 1∶2000 dilution) was added and incubated at room temperature for two hours. Cells were counterstained with DAPI (1 µg/ml) to visualize the nucleus.

## Supporting Information

Figure S1 SMO-1 interaction is specific to NHR-25. (A) Yeast two-hybrid analysis of the indicated proteins fused to the Gal4 activation domain (AD) or DNA binding domain (DB). Empty vector (No insert) controls are shown. β-galactosidase (LacZ) and HIS3 (3AT; 3-aminotriazole) reporters were assayed, and yeast viability was confirmed by growth on a plate lacking leucine and tryptophan (-Leu-Trp). Both NHR-25β and NHR-31 displayed self-activation activity, precluding analysis of their interactions with any of the AD fusions. (B) Due to the size of the matrix, the strains were plated on two plates. To rule out variation between plates, a negative control (i; AD and DB empty vectors) and two positive controls (RFS-1 interaction with RAD-51 (ii) and R01H10.5 (iii), respectively) are provided for each plate.
(TIF)

Figure S2 *smo-1(lf)* and *smo-1(lf); nhr-25(RNAi)* cause defects in 2° cell fate. (A) *Pegl-17::YFP* expression in vulval cells at the 4-cell stage (1° cell fate marker) in the animals of the indicated genotypes. Ectopically high expression of *Pegl-17::YFP* was observed in 2° fated cells in *smo-1(ok359)* and *smo-1(ok359)*; *nhr-25(RNAi)* animals. (B) The *egl-17::YFP* vulva marker is expressed in *smo-1 (lf)*-induced multivulva. Wild type expression of *egl-17::YFP* seen in vulD (a) and vulC (b) in late vulva morphogenesis. In *smo-1 (ok359)* and *smo-1 (ok359); nhr-25 (RNAi)* backgrounds (c and d), Muv is induced and the 1°/2° vulva marker *egl-17::GFP* is ectopically expressed. * indicates ectopic vulvae. (C) *egl-17* has been reported to be expressed in all Pn.p cells [34]. NHR-25, NHR-25(3KR) and SMO-1 were driven by an *egl-17* promoter for *in vivo* overexpression (Figure 8) from a vector carrying a polycistronic mCherry marker. We observed mCherry expression in Pn.p cells, indicating that this promoter is active in these cells. A representative image of mCherry expression in P3.p and P6.p cells from an *[egl-17::NHR-25(3KR)_polycistronic_mCherry]* transgenic animal is provided.
(TIF)

Figure S3 SMO-1 expression is required for NHR-25 to interact with UBC-9. (A) Indicated proteins were fused to the Gal4 activation domain (AD) or DNA binding domain (DB). Empty vector (No insert) controls are shown. (A) Yeast two-hybrid data confirmed that the SMO-1 V31K β-sheet mutation still binds to UBC-9, which indicated that the mutation did not disrupt the protein. The SMO-1 di-glycine deletion (ΔGG) prevented the interaction with UBC-9. (B) Yeast three-hybrid analysis. The indicated AD and DB fusions were expressed along with the pAG416 low copy yeast expression vector carrying either no insert or SMO-1. β-galactosidase staining is provided in A and B.
(TIF)

Figure S4 Confirmation of activity of sumoylation enzymes. *In vitro* sumoylation reactions were resolved by SDS-PAGE and visualized by Coomassie staining (A,B) or anti-NHR-25 immunoblotting (C). (A and B) used a recombinant SF-1 partial hinge-LBD fragment as a substrate and (C) used a recombinant 6×His-MBP-NHR-25 (amino acids 161–541) fragment. All reactions used recombinant hE1. In (A), hE2 (UBC9) and hSUMO1 were used. (B and C) used CeUBC-9 and CeSMO-1. Recombinant hSENP1 SUMO protease was included in each experiment to demonstrate that bands reflected sumoylated species. A size standard in kilodaltons (kDa) is provided.
(TIF)

Figure S5 The NHR-25 hinge domain is sumoylated *in vitro* on three lysines. *In vitro* sumoylation reactions were resolved by SDS-PAGE and visualized by anti-NHR-25 immunoblotting (A) or Coomassie staining (B). Both reactions used hE1, hE2, hSUMO1, and a recombinant NHR-25 substrate (6×His-MBP-NHR-25 (amino acids 161–541)). In (A) recombinant hSENP1 SUMO protease was included. In (B), the substrates were wild type NHR-25 (WT) and NHR-25 2KR (K170R K236R) and NHR-25 3KR (K165 K170R K236R) mutants where SUMO acceptor lysines were mutated to arginine. A size standard in kilodaltons (kDa) is provided.
(TIF)

Figure S6 Sumo1 and SMO-1 do not readily form poly-SUMO chains. (A) Anti-NHR-25 immunoblots on sumoylation reactions incubated for the indicated number of hours. E1 enzyme was incubated with the indicated E2 and SUMO combinations. Methyl-hSUMO1 is a modified protein that blocks SUMO chain formation. The asterisk (*) indicates a non-specific band in the NHR-25 substrate control lane (no sumoylation enzymes added). NHR-25 isoforms predicted to contain one, two, and three SUMO proteins covalently attached are indicated (1-Su, 2-Su, 3-Su, respectively). (B–E) Short course sumoylation time courses using hE1, hE2, and hSUMO1 (B,D) or hE1, CeUBC-9, and CeSMO-1 (C,E). The substrate was recombinant 6×His-MBP-NHR-25 (amino acids 161–541). Reaction time in minutes, and a size standard in kilodaltons (kDa) are provided. The final lane is a substrate only control. Coomassie stained polyacrylamide gels (B, C) and anti-NHR-25 immunoblots (D,E) are shown.
(TIF)

Figure S7 Sumoylation affects NHR-25 binding to canonical SF-1 sites. (A) Sequence of binding sites used in the Y1H and EMSA experiments. The mutation in the MIS binding site (MIS MUT) is underlined. The canonical binding site of the NHR-25 ortholog, SF-1, is 5′-YCAAGGYCR-3′ (Y = T/C, R = G/A) [63]. (B) Y1H analysis. Two tandem copies of the indicated binding sites upstream of a LacZ reporter were integrated into the YM4271 yeast strain. Indicated proteins were fused to the Gal4 activation domain (AD). (C) EMSA data. Annealed oligonucleotides carrying the MIS WT, MIS MUT, and CYP11A1 binding sites were incubated with: sumoylation enzymes (hUbc9+CeSMO-1) with or without hE1 enzyme, and NHR-25 DBD substrate. Recombinant hSENP1 SUMO protease was included to demonstrate that bands reflected sumoylated species. (D) EMSA analysis of NHR-25 binding to annealed oligonucleotides carrying both MIS and CYP11A1 binding sites (2×NR5RE). Increasing amounts of sumoylated NHR-25 DBD were added to 1 µM of annealed oligos (200–700 nM NHR-25 in 100 nM increments). Both wild-type (WT) and mutated (MUT) binding sites were analyzed. (E) EMSAs were performed on the 2×NR5RE in which the NHR-25 DBD was sumoylated at 37°C for the indicated time. (C–E) The corresponding proteins in the EMSA were detected by anti-MBP immunoblotting (input). The positions of unsumoylated and sumoylated NHR-25 DBD are indicated.
(TIF)

Table S1 Overexpression of NHR-25 in hyp7 or seam cells does not cause Muv induction. Table providing scoring of overall multivulva (Muv) induction in the indicated strains/genotypes, as well as induction in individual VPCs. Number of animals (n) scored for each strain genotype is provided. Use of brackets denotes transgenic genotypes.
(DOCX)

Table S2 Sequences of oligonucleotides and gBlocks used in this study. All sequences are displayed in a 5′ to 3′ orientation. (A) Primers used to clone the indicated cDNAs and promoters. Sequences of the attB recombination sites and Myc and FLAG epitopes are indicated as described in the table. (B) Sequences of the primers used to generate the indicated mutations by site-directed mutagenesis. (C) gBlocks used in this study. NR5 binding sites and minimal promoters are indicated as described in the table. (D) Sequences of oligonucleotides from SF-1 target gene promoters used in EMSA assays are shown. m, mouse; h, human; MIS, Mullerian Inhibiting Substance; ; CYP11A1, Cytochrome P450, Family 11, Subfamily A, Polypeptide 1; 2×NR5RE, nuclear receptor NR5 family Response Element. The NR5RE oligos carry an mMIS and hCYP11A1 binding site. SF-1 binding site is highlighted in bold.
(DOCX)

Table S3 Plasmids generated for this study. The vector backbones used for Gateway cloning are provided, as is a description of each vector. pDONR221 (Invitrogen) and pDONR-P4P1r (Invitrogen) are entry vectors for cDNA and promoter cloning, respectively. pAD and pDB are Y2H vectors for generating N-terminal fusions of the Gal4 activation domain (AD) and DNA binding domain (DB), respectively, to proteins of interest [18]. pMW2 and pMW3 are reporter vectors for Y1H assays [49]. pMW2 is used to clone DNA fragments upstream of a HIS3 reporter gene, pMW3 is used to clone DNA fragments upstream of a LacZ reporter. pETG10A is used to generate N-terminal 6×His fusions for bacterial expression. pETG41A is used to generate N-terminal 6×His-MBP fusions for bacterial expression. pDEST-CMV-Myc is used to generate N-terminal Myc fusions under the control of a CMV promoter for mammalian cell expression. pKA921 is used for two-fragment Gateway cloning to create promoter-cDNA combinations. A polycistronic mCherry cassette with an *unc-54* 3′-UTR marks the tissues where the array is expressed. pCFJ150 is used to generate *C. elegans* expression vectors through three-fragment Gateway cloning [51]. pAG415 GAL-ccbB is used for constitutive expression of cDNAs in yeast [50]. pET-DUET1 (Novagen) is used for simultaneous expression of two cDNAs in bacteria.
(DOCX)
