# Supplementary figures and images for: Sumoylated NHR-25/NR5A Regulates Cell Fate during C. elegans Vulval Development
Source: PLoS Genet. 2013 Dec 12;9(12):e1003992. doi: 10.1371/journal.pgen.1003992 (PMC3861103; doi:10.1371/journal.pgen.1003992)

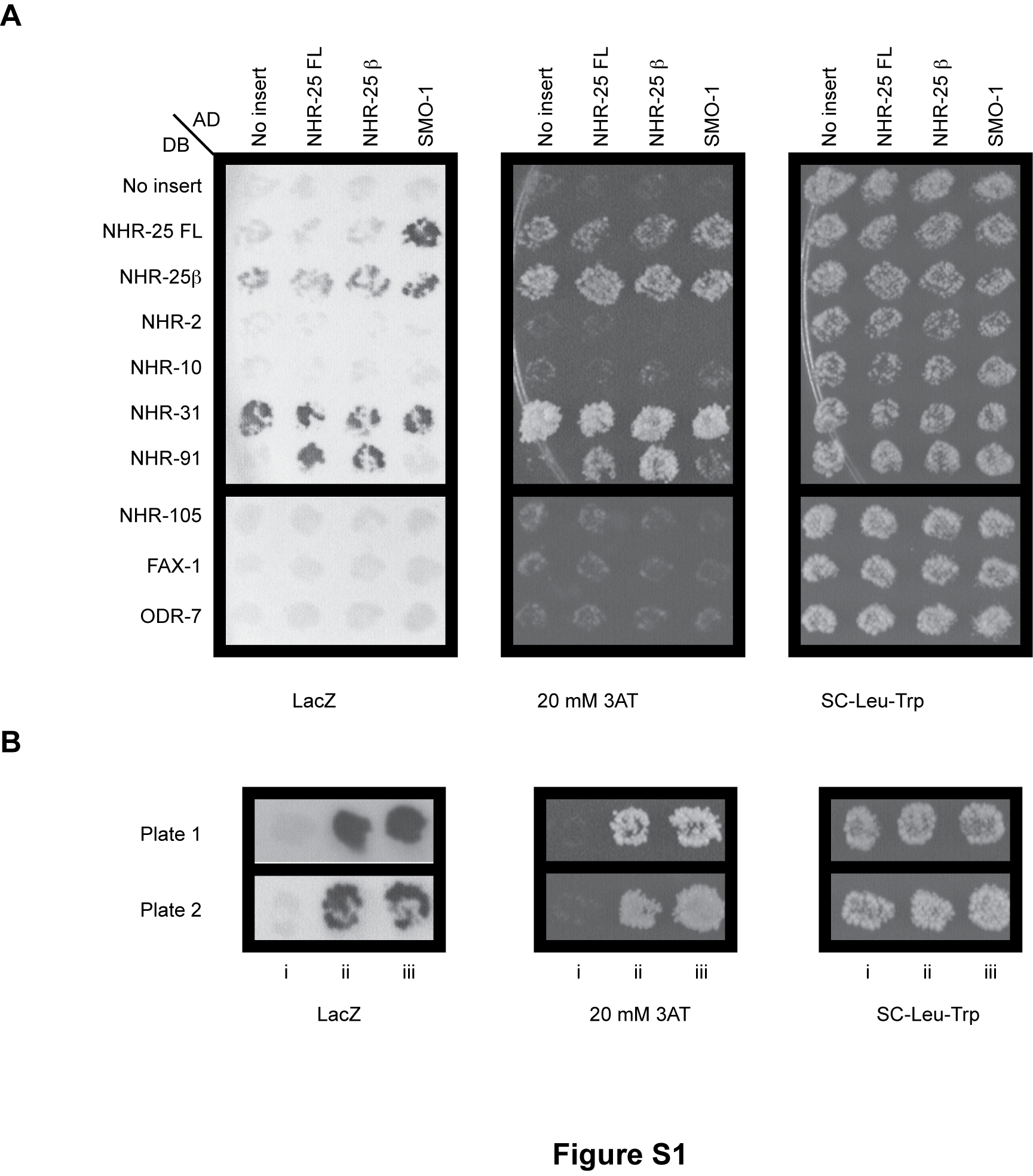

Supplement: Figure S1 — SMO-1 interaction is specific to NHR-25. (A) Yeast two-hybrid analysis of the indicated proteins fused to the Gal4 activation domain (AD) or DNA binding domain (DB). Empty vector (No insert) controls are shown. β-galactosidase (LacZ) and HIS3 (3AT; 3-aminotriazole) reporters were assayed, and yeast viability was confirmed by growth on a plate lacking leucine and tryptophan (-Leu-Trp). Both NHR-25β and NHR-31 displayed self-activation activity, precluding analysis of their interactions with any of the AD fusions. (B) Due to the size of the matrix, the strains were plated on two plates. To rule out variation between plates, a negative control (i; AD and DB empty vectors) and two positive controls (RFS-1 interaction with RAD-51 (ii) and R01H10.5 (iii), respectively) are provided for each plate. (TIF) [file pgen.1003992.s001.tif]

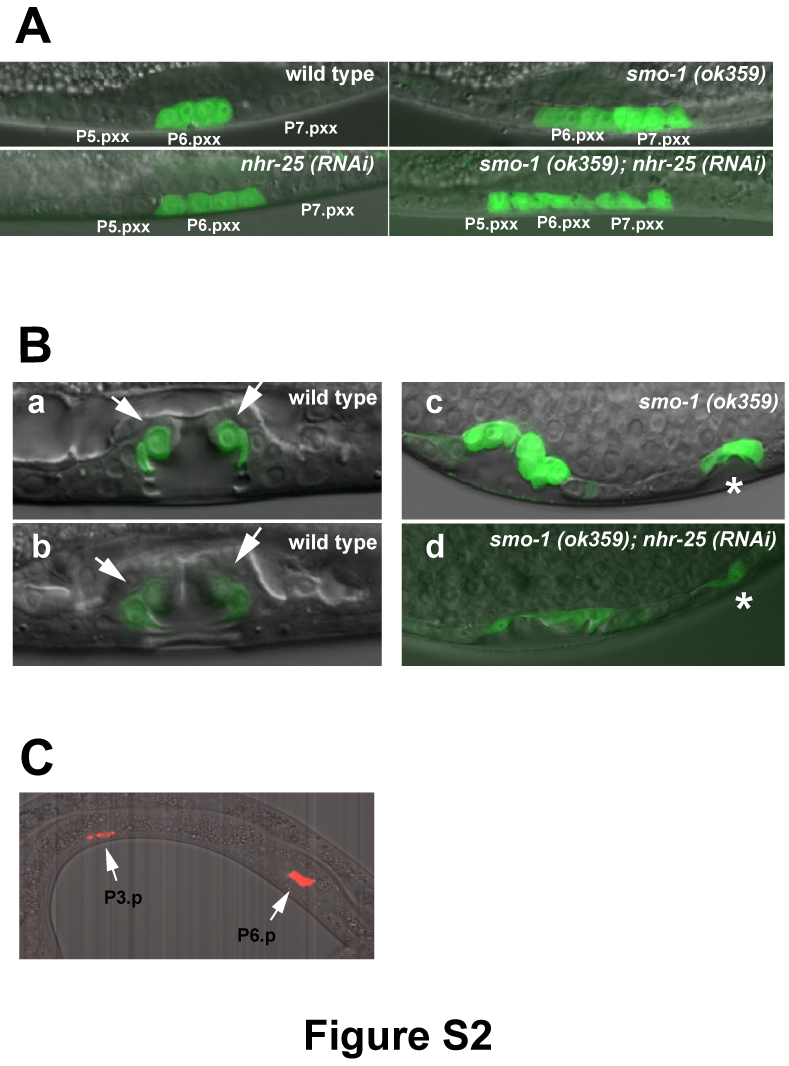

Supplement: Figure S2 — smo-1(lf) and smo-1(lf); nhr-25(RNAi) cause defects in 2° cell fate. (A) Pegl-17::YFP expression in vulval cells at the 4-cell stage (1° cell fate marker) in the animals of the indicated genotypes. Ectopically high expression of Pegl-17::YFP was observed in 2° fated cells in smo-1(ok359) and smo-1(ok359); nhr-25(RNAi) animals. (B) The egl-17::YFP vulva marker is expressed in smo-1 (lf)-induced multivulva. Wild type expression of egl-17::YFP seen in vulD (a) and vulC (b) in late vulva morphogenesis. In smo-1 (ok359) and smo-1 (ok359); nhr-25 (RNAi) backgrounds (c and d), Muv is induced and the 1°/2° vulva marker egl-17::GFP is ectopically expressed. * indicates ectopic vulvae. (C) egl-17 has been reported to be expressed in all Pn.p cells [34]. NHR-25, NHR-25(3KR) and SMO-1 were driven by an egl-17 promoter for in vivo overexpression (Figure 8) from a vector carrying a polycistronic mCherry marker. We observed mCherry expression in Pn.p cells, indicating that this promoter is active in these cells. A representative image of mCherry expression in P3.p and P6.p cells from an [egl-17::NHR-25(3KR)_polycistronic_mCherry] transgenic animal is provided. (TIF) [file pgen.1003992.s002.tif]

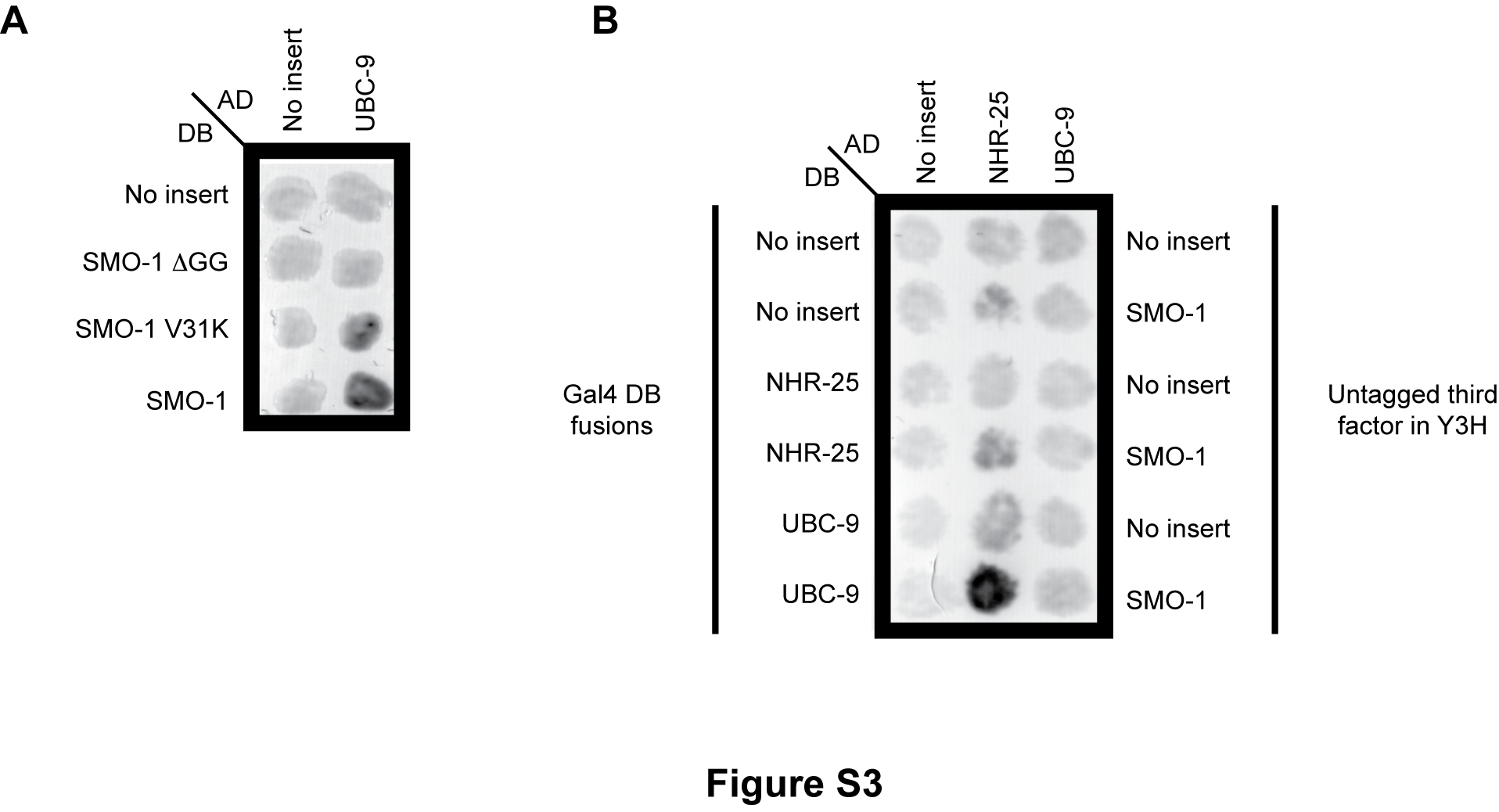

Supplement: Figure S3 — SMO-1 expression is required for NHR-25 to interact with UBC-9. (A) Indicated proteins were fused to the Gal4 activation domain (AD) or DNA binding domain (DB). Empty vector (No insert) controls are shown. (A) Yeast two-hybrid data confirmed that the SMO-1 V31K β-sheet mutation still binds to UBC-9, which indicated that the mutation did not disrupt the protein. The SMO-1 di-glycine deletion (ΔGG) prevented the interaction with UBC-9. (B) Yeast three-hybrid analysis. The indicated AD and DB fusions were expressed along with the pAG416 low copy yeast expression vector carrying either no insert or SMO-1. β-galactosidase staining is provided in A and B. (TIF) [file pgen.1003992.s003.tif]

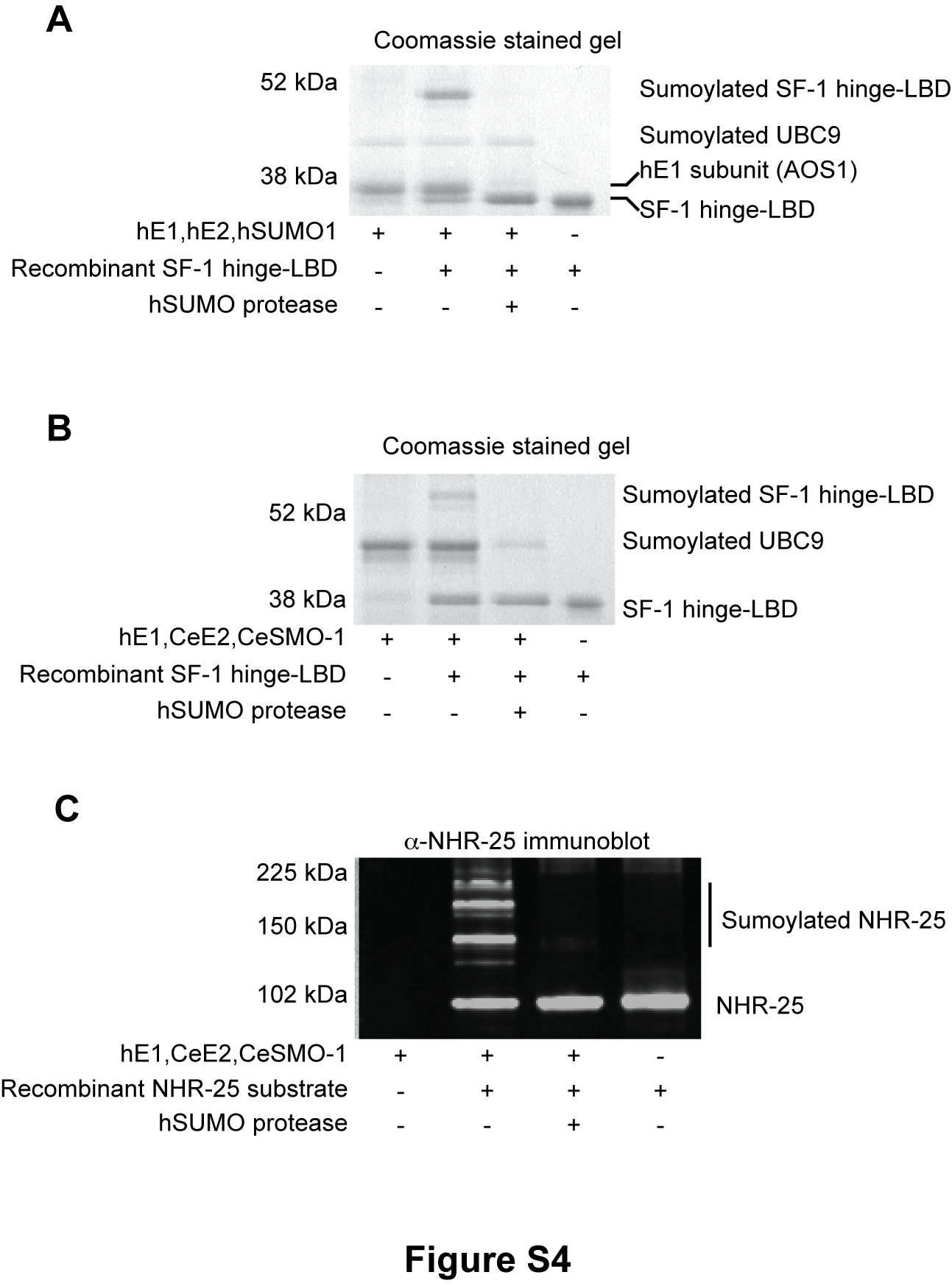

Supplement: Figure S4 — Confirmation of activity of sumoylation enzymes. In vitro sumoylation reactions were resolved by SDS-PAGE and visualized by Coomassie staining (A,B) or anti-NHR-25 immunoblotting (C). (A and B) used a recombinant SF-1 partial hinge-LBD fragment as a substrate and (C) used a recombinant 6×His-MBP-NHR-25 (amino acids 161–541) fragment. All reactions used recombinant hE1. In (A), hE2 (UBC9) and hSUMO1 were used. (B and C) used CeUBC-9 and CeSMO-1. Recombinant hSENP1 SUMO protease was included in each experiment to demonstrate that bands reflected sumoylated species. A size standard in kilodaltons (kDa) is provided. (TIF) [file pgen.1003992.s004.tif]

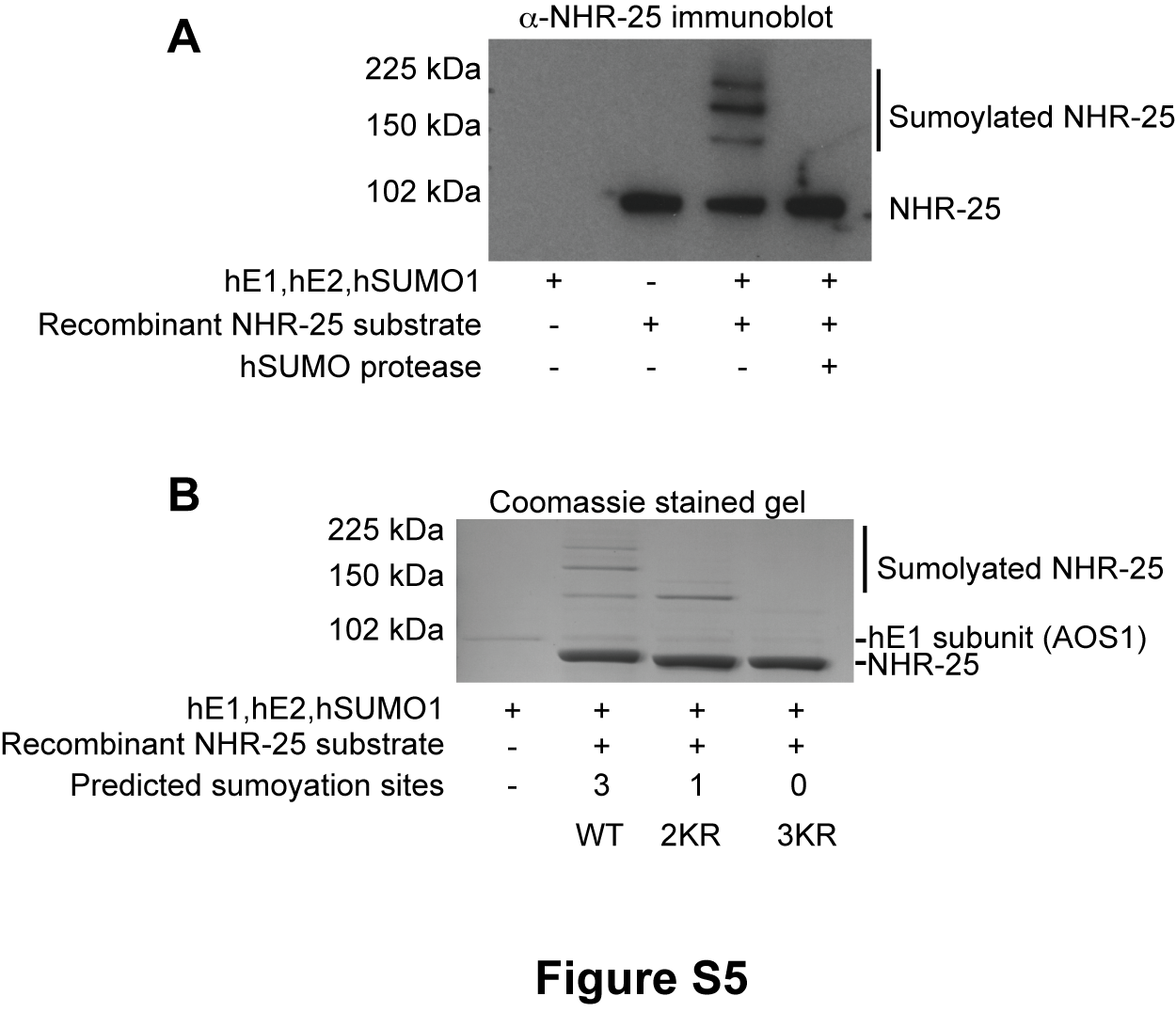

Supplement: Figure S5 — The NHR-25 hinge domain is sumoylated in vitro on three lysines. In vitro sumoylation reactions were resolved by SDS-PAGE and visualized by anti-NHR-25 immunoblotting (A) or Coomassie staining (B). Both reactions used hE1, hE2, hSUMO1, and a recombinant NHR-25 substrate (6×His-MBP-NHR-25 (amino acids 161–541)). In (A) recombinant hSENP1 SUMO protease was included. In (B), the substrates were wild type NHR-25 (WT) and NHR-25 2KR (K170R K236R) and NHR-25 3KR (K165 K170R K236R) mutants where SUMO acceptor lysines were mutated to arginine. A size standard in kilodaltons (kDa) is provided. (TIF) [file pgen.1003992.s005.tif]

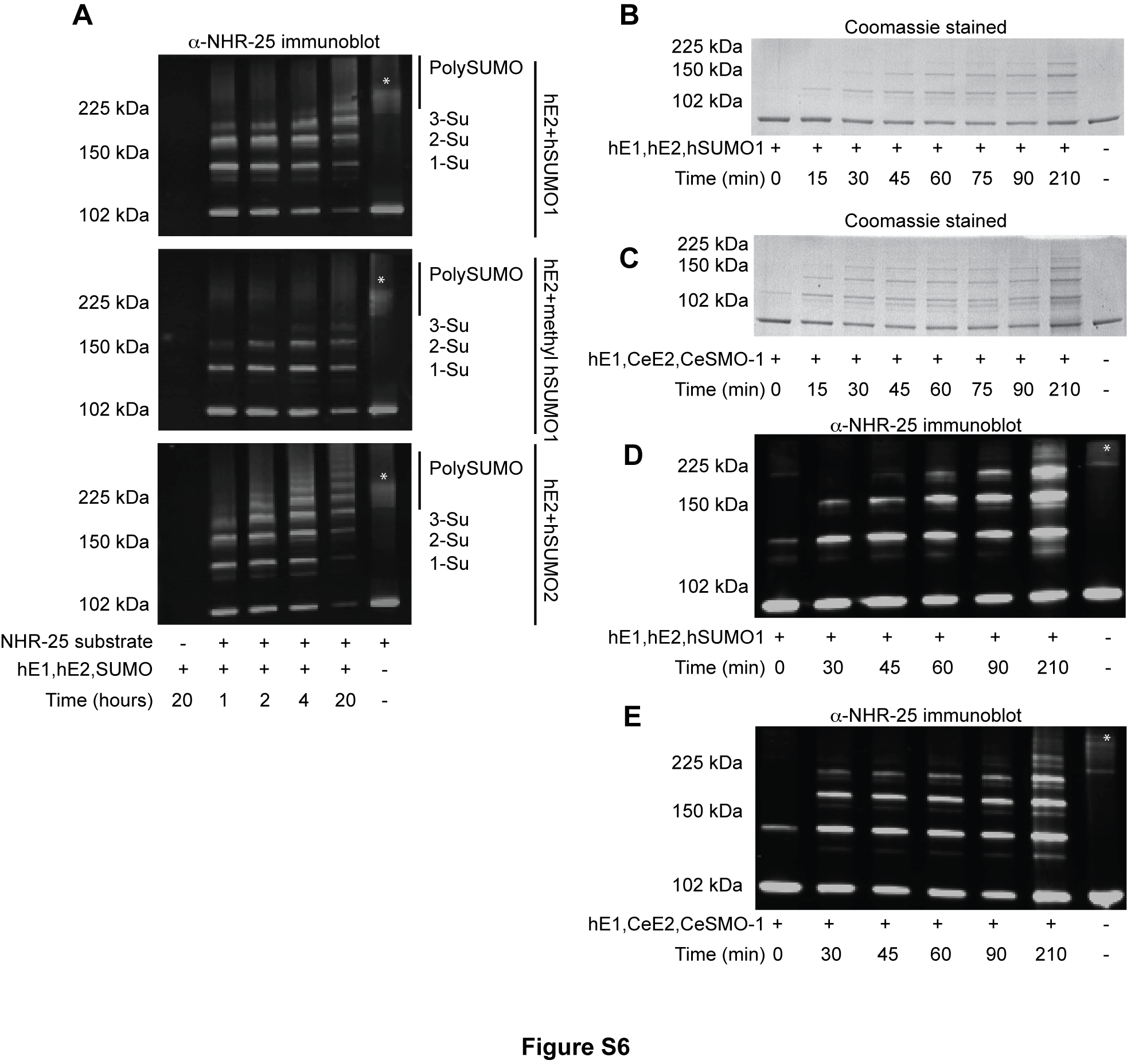

Supplement: Figure S6 — Sumo1 and SMO-1 do not readily form poly-SUMO chains. (A) Anti-NHR-25 immunoblots on sumoylation reactions incubated for the indicated number of hours. E1 enzyme was incubated with the indicated E2 and SUMO combinations. Methyl-hSUMO1 is a modified protein that blocks SUMO chain formation. The asterisk (*) indicates a non-specific band in the NHR-25 substrate control lane (no sumoylation enzymes added). NHR-25 isoforms predicted to contain one, two, and three SUMO proteins covalently attached are indicated (1-Su, 2-Su, 3-Su, respectively). (B–E) Short course sumoylation time courses using hE1, hE2, and hSUMO1 (B,D) or hE1, CeUBC-9, and CeSMO-1 (C,E). The substrate was recombinant 6×His-MBP-NHR-25 (amino acids 161–541). Reaction time in minutes, and a size standard in kilodaltons (kDa) are provided. The final lane is a substrate only control. Coomassie stained polyacrylamide gels (B, C) and anti-NHR-25 immunoblots (D,E) are shown. (TIF) [file pgen.1003992.s006.tif]

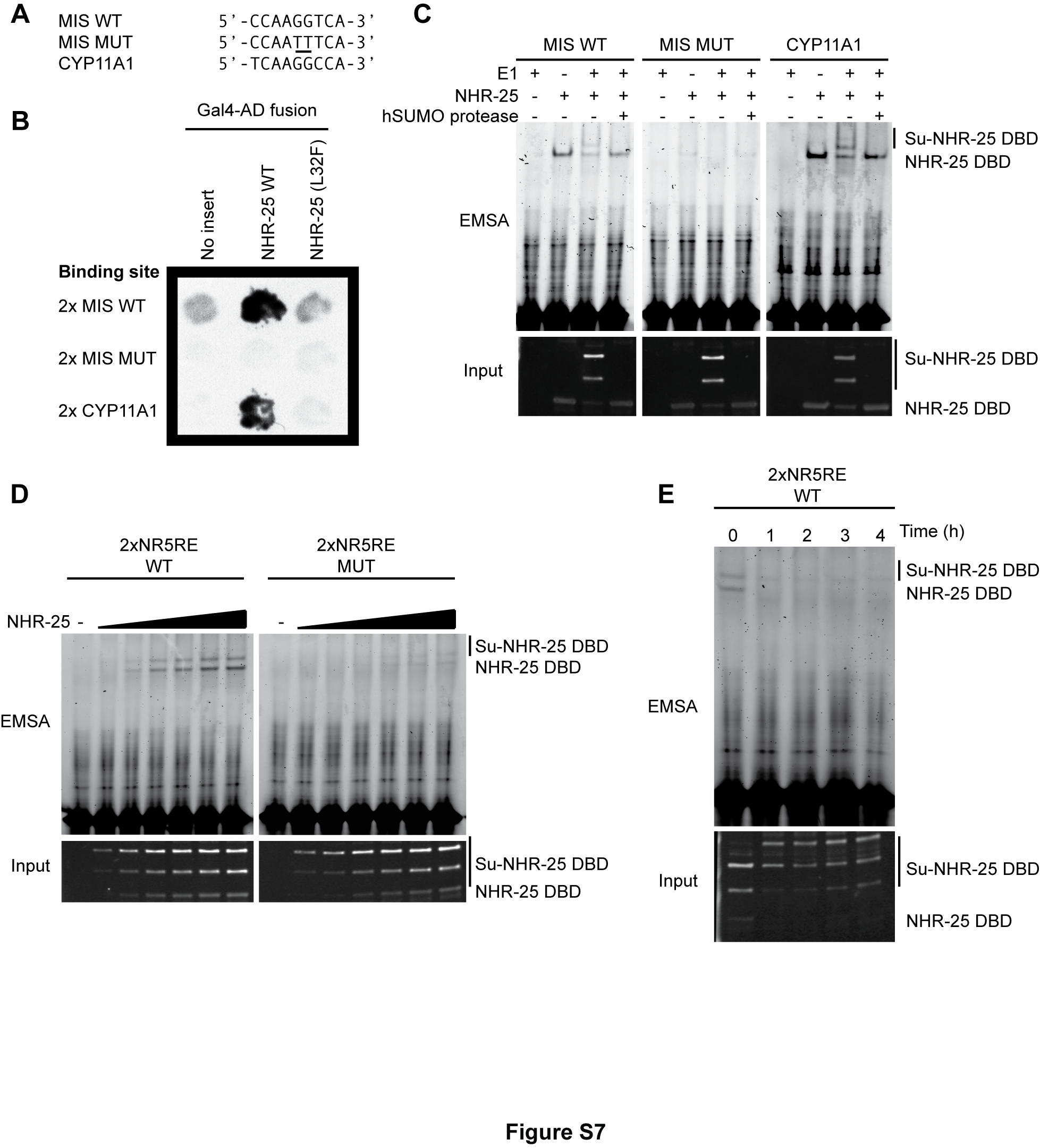

Supplement: Figure S7 — Sumoylation affects NHR-25 binding to canonical SF-1 sites. (A) Sequence of binding sites used in the Y1H and EMSA experiments. The mutation in the MIS binding site (MIS MUT) is underlined. The canonical binding site of the NHR-25 ortholog, SF-1, is 5′-YCAAGGYCR-3′ (Y = T/C, R = G/A) [63]. (B) Y1H analysis. Two tandem copies of the indicated binding sites upstream of a LacZ reporter were integrated into the YM4271 yeast strain. Indicated proteins were fused to the Gal4 activation domain (AD). (C) EMSA data. Annealed oligonucleotides carrying the MIS WT, MIS MUT, and CYP11A1 binding sites were incubated with: sumoylation enzymes (hUbc9+CeSMO-1) with or without hE1 enzyme, and NHR-25 DBD substrate. Recombinant hSENP1 SUMO protease was included to demonstrate that bands reflected sumoylated species. (D) EMSA analysis of NHR-25 binding to annealed oligonucleotides carrying both MIS and CYP11A1 binding sites (2×NR5RE). Increasing amounts of sumoylated NHR-25 DBD were added to 1 µM of annealed oligos (200–700 nM NHR-25 in 100 nM increments). Both wild-type (WT) and mutated (MUT) binding sites were analyzed. (E) EMSAs were performed on the 2×NR5RE in which the NHR-25 DBD was sumoylated at 37°C for the indicated time. (C–E) The corresponding proteins in the EMSA were detected by anti-MBP immunoblotting (input). The positions of unsumoylated and sumoylated NHR-25 DBD are indicated. (TIF) [file pgen.1003992.s007.tif]
