# Supplementary material for: Sumoylated NHR-25/NR5A Regulates Cell Fate during C. elegans Vulval Development
Source: PLoS Genet. 2013 Dec 12;9(12):e1003992. doi: 10.1371/journal.pgen.1003992 (PMC3861103; doi:10.1371/journal.pgen.1003992)
Supplement: Table S1 — Overexpression of NHR-25 in hyp7 or seam cells does not cause Muv induction. Table providing scoring of overall multivulva (Muv) induction in the indicated strains/genotypes, as well as induction in individual VPCs. Number of animals (n) scored for each strain genotype is provided. Use of brackets denotes transgenic genotypes. (DOCX) [file pgen.1003992.s008.docx]

**Table S1: Overexpression of NHR-25 in hyp7 or seam cells does not cause Muv induction**

| **Genotype/Strain** | **Muv (%)** | **Pn.p induction (%)** | | | | | | **n** |
| --- | --- | --- | --- | --- | --- | --- | --- | --- |
|  |  | **P3.p** | **P4.p** | **P5.p** | **P6.p** | **P7.p** | **P8.p** |  |
| wild type | 0 | 0 | 0 | 100 | 100 | 100 | 0 | 60 |
| *nhr-25(RNAi)* | 0 | 0 | 0 | 98 | 100 | 100 | 0 | 79 |
| *smo-1(RNAi)* | 12.5 | 0 | 0 | 100 | 100 | 100 | 12.5 | 16 |
| *[Pnhr-25::NHR-25::GFP]* |  |  |  |  |  |  |  |  |
| OP33 | 0 | 0 | 0 | 100 | 100 | 100 | 0 | 45 |
| OP33*; smo-1(RNAi)* | 30 | 0 | 10 | 100 | 100 | 100 | 20 | 10 |
| *[Pegl-17::NHR-25]* |  |  |  |  |  |  |  |  |
| HL102 | 8.5 | 0 | 0 | 100 | 100 | 100 | 8.5 | 23 |
| *[Pegl-17::NHR-25(3KR)]* |  |  |  |  |  |  |  |  |
| HL107 | 18 | 4.5 | 4.5 | 82 | 100 | 86 | 9 | 22 |
| HL108 | 18.5 | 0 | 7.4 | 78 | 96 | 85 | 11 | 27 |
| HL110 | 22 | 5.5 | 5 | 89 | 100 | 89 | 11 | 18 |
| *[Pegl-17::SMO-1]* |  |  |  |  |  |  |  |  |
| HL117 | 0 | 0 | 0 | 100 | 100 | 100 | 0 | 99 |
| *[Pgrl-21::NHR-25]* |  |  |  |  |  |  |  |  |
| HL111 | 0 | 0 | 0 | 100 | 100 | 100 | 0 | 20 |
| HL112 | 0 | 0 | 0 | 100 | 100 | 100 | 0 | 26 |
| *[Pgrl-21::SMO-1]* |  |  |  |  |  |  |  |  |
| HL121 | 0 | 0 | 0 | 100 | 100 | 100 | 0 | 44 |
| *[Pwrt-2::NHR-25]* |  |  |  |  |  |  |  |  |
| HL113 | 0 | 0 | 0 | 100 | 100 | 100 | 0 | 20 |
| HL114 | 0 | 0 | 0 | 100 | 100 | 100 | 0 | 32 |
| *[Pwrt-2::SMO-1]* |  |  |  |  |  |  |  |  |
| HL115 | 0 | 0 | 0 | 100 | 100 | 100 | 0 | 20 |
| HL116 | 0 | 0 | 0 | 100 | 100 | 100 | 0 | 46 |
