# Supplementary material for: Sumoylated NHR-25/NR5A Regulates Cell Fate during C. elegans Vulval Development
Source: PLoS Genet. 2013 Dec 12;9(12):e1003992. doi: 10.1371/journal.pgen.1003992 (PMC3861103; doi:10.1371/journal.pgen.1003992)
Supplement: Table S2 — Sequences of oligonucleotides and gBlocks used in this study. All sequences are displayed in a 5′ to 3′ orientation. (A) Primers used to clone the indicated cDNAs and promoters. Sequences of the attB recombination sites and Myc and FLAG epitopes are indicated as described in the table. (B) Sequences of the primers used to generate the indicated mutations by site-directed mutagenesis. (C) gBlocks used in this study. NR5 binding sites and minimal promoters are indicated as described in the table. (D) Sequences of oligonucleotides from SF-1 target gene promoters used in EMSA assays are shown. m, mouse; h, human; MIS, Mullerian Inhibiting Substance; ; CYP11A1, Cytochrome P450, Family 11, Subfamily A, Polypeptide 1; 2×NR5RE, nuclear receptor NR5 family Response Element. The NR5RE oligos carry an mMIS and hCYP11A1 binding site. SF-1 binding site is highlighted in bold. (DOCX) [file pgen.1003992.s009.docx]

**Table S2. Sequences of oligonucleotides and gBlocks used in this study.**

**A) Primers for generating Gateway entry clones (attB sites are capitalized)**

Primers for cloning full-length *nhr-25* cDNA (no start codon for N-terminal fusions; stop codon)

GGGGACAAGTTTGTACAAAAAAGCAGGCTGGttgactgacgtcgagaggatg

GGGGACCACTTTGTACAAGAAAGCTGGGTCCTAttatgatgccatgtacggcac

Primer for Gateway cloning FLAG-tagged cDNAs

GGGGACAAGTTTGTACAAAAAAGCAGGCTGGgattacaaggatgacgatgacaag

Primers for generating FLAG-*nhr-25* cDNA (amino acids 161-541; FLAG tag is underlined)

gattacaaggatgacgacgataagcaaactaaaatcaaaactgaa

GGGGACCACTTTGTACAAGAAAGCTGGGTCCTAcgatcggttgacagtagtgc

Primer for introducing FLAG tag and *K165R* mutation in *nhr-25* cDNA (amino acids 161-541; FLAG tag is underlined)

gattacaaggatgacgacgataagcaaactaaaatcagaactgaa

Primers for cloning *nhr-25* cDNA (amino acids 1-173; no start codon for N-terminal fusions)

GGGGACAAGTTTGTACAAAAAAGCAGGCTGGttgactgacgtcgagaggatg

GGGGACCACTTTGTACAAGAAAGCTGGGTCCTAatactcagttttgatatattcagt

Primers for cloning full-length *nhr-91* cDNA (no start codon for N-terminal fusions; no stop codon for C-terminal fusions)

GGGGACAAGTTTGTACAAAAAAGCAGGCTtggacgttgcggactcgacat

GGGGACCACTTTGTACAAGAAAGCTGGGtactcgtcgaacactagac

Primers for cloning full-length *smo-1* cDNA (no start codon for N-terminal fusions; stop codon)

GGGGACAAGTTTGTACAAAAAAGCAGGCttggccgatgatgcagctcaa

GGGGACCACTTTGTACAAGAAAGCTGGGctagaatccgcccagctgctctt

Primers for cloning *smo-1ΔF* (ends in GG) cDNA (no start codon for N-terminal fusions)

GGGGACAAGTTTGTACAAAAAAGCAGGCttggccgatgatgcagctcaa

GGGGACCACTTTGTACAAGAAAGCTGGGTCCTActatccgcccagctgctcttg

Primers for cloning *smo-1ΔGG* cDNA (sumoylation-defective; no start codon for N-terminal fusions)

GGGGACAAGTTTGTACAAAAAAGCAGGCttggccgatgatgcagctcaa

GGGGACCACTTTGTACAAGAAAGCTGGGctacagctgctcttggtagacc

Primer for fusing Myc tag onto *nhr-25* cDNA 5’ end (Myc tag is underlined)

gcatcaatgcagaagctgatctcagaggaggacctgatgactgacgtcgagaggatg

Primers for cloning Myc-*nhr-25* cDNA

GGGGACAAGTTTGTACAAAAAAGCAGGCTGGgcatcaatgcagaagctgatctcagagga

GGGGACCACTTTGTACAAGAAAGCTGGGTCCTAttatgatgccatgtacggcac

Primers for adding TEV protease cleavage site to *Ceubc-9* cDNA

AAAACTTGTATTTCCAGGGCatgtcgggaattgctgcagg

GGGGACCACTTTGTACAAGAAAGCTGGGTCCTActcgagcatttgcttctggac

Primers for cloning TEV-*Ceubc-9* cDNA

GGGGACAAGTTTGTACAAAAAAGCAGGCTGGggcagagaaaacttgtatttccagggc

GGGGACCACTTTGTACAAGAAAGCTGGGTCCTActcgagcatttgcttctggac

Primers for cloning NLS-3xVenus

GGGGACAAGTTTGTACAAAAAAGCAGGCTGGatgactgctccaaagaagaag

GGGACCACTTTGTACAAGAAAGCTGGGTCCTActtacaattctacgaatgcta

Primers for cloning 8xNR5RE promoter fragments

GGGGACAACTTTGTATAGAAAAGTTGattaaccctcactaaaggga

GGGGACTGCTTTTTTGTACAAACTTGTCattttttctgagctcggtaccct

Primers for cloning *egl-17* promoter

GGGGACAACTTTGTATAGAAAAGTTGgcatttctttttccattaa

GGGGACTGCTTTTTTGTACAAACTTGTCatagctcacatttcgggca

**B) Primers for site-directed mutagenesis**

Primers for making *smo-1 V31K* mutant cDNA

gcaacgaagtgcacttccgtaaaaagtatggaacctctatgg

ccatagaggttccatactttttacggaagtgcacttcgttgc

Primers for making *nhr-25 L32F* mutant cDNA

ctggatatcactacggctttctgacgtgtgaaagt

actttcacacgtcagaaagccgtagtgatatccag

Primers for making *nhr-25 K84R* mutant cDNA

cctcacaatgggaatgagaatggaagcggtgcgtg

cacgcaccgcttccattctcattcccattgtgagg

Primers for making *nhr-25 K165R* mutant cDNA

aaatcaactattttgaccaaactaaaatcagaactgaatatatcaaaactgagtatga

tcatactcagttttgatatattcagttctgattttagtttggtcaaaatagttgattt

Primers for making *nhr-25 K170R* mutant cDNA

ctaaaatcaaaactgaatatatcagaactgagtatgatgcacatctcca

tggagatgtgcatcatactcagttctgatatattcagttttgattttag

Primers for making *nhr-25 K236R* mutant cDNA

gcagcttatcagttgaatgaagtcagacaggagccattt

aaatggctcctgtctgacttcattcaactgataagctgc

Primers for making *nhr-25 K165R K170* mutant cDNA

aaatcaactattttgaccaaactaaaatcagaactgaatatatcagaactgagtatga

tcatactcagttctgatatattcagttctgattttagtttggtcaaaatagttgattt

Primers for making *nhr-25 E167A E172A* mutant cDNA

aaactgcatatatcaaaactgaatatatcaaaactgcgtatgatgcacatctccagagtc

tcatacgcagttttgatatattcagttttgatatatgcagttttgattttagtttggtca

Primers for making *nhr-25 E238A* mutant cDNA

acaggcgccatttgactattc

tggcgcctgtttgacttcattcaac

Primers for making *nhr-25 K170R* (in amino acids 161-541 vector) mutant cDNA

caaactaaaatcagaactgaatatatcaa

cttatcgtcgtcatccttgtaatc

**C) gBlock sequences used**

2x MIS gBlocks (for Y1H)

GGGGCAACTTTGTATAGAAAAGTTGccaaggtcatagcggccagccaaggtcacaagttt

GGGGCTGCTTTTTTGTACAAACTTGTGaccttggctggccgctatgaccttggcaacttt

2x MIS Mutated gBlock (For Y1H)

GGGGCAACTTTGTATAGAAAAGTTGccaatttcatagcggccagccaatttcacaagttt

GGGGCTGCTTTTTTGTACAAACTTGTGaaattggctggccgctatgaaattggcaacttt

2x CYP11A1 site gBlock (For Y1H)

GGGGCAACTTTGTATAGAAAAGTTGtcaaggccaagggcgacagtcaaggccacaagttt

GGGGCTGCTTTTTTGTACAAACTTGTggccttgactgtcgcccttggccttgacaacttt

8xNR5RE(WT)-pes-10Δ (MIS sites in green, CYP11A1 sites in blue, pes-10Δ minimal promoter in yellow)

attaaccctcactaaagggagccaaggtcatagcggccagtcaaggccaagggcgacagccaaggtcaggttccgcggtcaaggccaaaatgaaataagcttgcatgccaaggtcaaagggcgacgtcaaggccagcgtcaatagccaaggtcatggtgagtagtcaaggccagaaccggcctgcaggatcgattttttgcaaattacgagcgttgtagggggcggagcgataggtcctataggttttggtatatcatcattcattcattcattggtacattcatttacccaccttcctctttctgagcttctctggagttctgtgcttcctttttcccttatctttatactgtaatttttaactttcaggcattgattggatccccgggattggccaaaggacccaaaggtatgtttcgaatgatactaacataacatagaacattttcaggaggacccttgcttggagggtaccgagctcagaaaaa

8xNR5RE(MUT)-pes-10Δ (MIS MUT sites in green, CYP11A1 MUT sites in blue, pes-10Δ minimal promoter in yellow; mutated bases underlined)

attaaccctcactaaagggagaaaaggtcatagcggccagtcaaaaccaagggcgacagccccggtcaggttccgcggggaaggccaaaatgaaataagcttgcatgccaaaatcaaagggcgacgtgggggccagcgtcaatagccaaaaacatggtgagtagttttggccagaaccggcctgcaggatcgattttttgcaaattacgagcgttgtagggggcggagcgataggtcctataggttttggtatatcatcattcattcattcattggtacattcatttacccaccttcctctttctgagcttctctggagttctgtgcttcctttttcccttatctttatactgtaatttttaactttcaggcattgattggatccccgggattggccaaaggacccaaaggtatgtttcgaatgatactaacataacatagaacattttcaggaggacccttgcttggagggtaccgagctcagaaaaa

**D) Primers for EMSAs (NR5 binding sites are in bold font)**

MIS WT

gatccgccaggcactgtccc**ccaaggtca**cctttggtgttgataagatccc

tcgagggatcttatcaacaccaaagg**tgaccttgg**gggacagtgcctggcg

MIS MUT

gatccgccaggcactgtccc**ccaatttca**cctttggtgttgataagatccc

tcgagggatcttatcaacaccaaagg**tgaaattgg**gggacagtgcctggcg

CYP11A1

acattttatcagcttctggta**tggccttga**gctggtagttataatcttggc

gccaagattataactaccagc**tcaaggcca**taccagaagctgataaaatgt

2xNR5RE WT

ctaaagggag**ccaaggtca**tagcggccag**tcaaggcca**agggcgacag

ctgtcgccct**tggccttga**ctggccgcta**tgaccttgg**ctccctttag

2xNR5RE MUT

ctaaagggag**aaaaggtca**tagcggccag**tcaaaacca**agggcgacag

ctgtcgccct**tggttttga**ctggccgcta**tgacctttt**ctccctttag
