# Supplementary material for: Sumoylated NHR-25/NR5A Regulates Cell Fate during C. elegans Vulval Development
Source: PLoS Genet. 2013 Dec 12;9(12):e1003992. doi: 10.1371/journal.pgen.1003992 (PMC3861103; doi:10.1371/journal.pgen.1003992)
Supplement: Table S3 — Plasmids generated for this study. The vector backbones used for Gateway cloning are provided, as is a description of each vector. pDONR221 (Invitrogen) and pDONR-P4P1r (Invitrogen) are entry vectors for cDNA and promoter cloning, respectively. pAD and pDB are Y2H vectors for generating N-terminal fusions of the Gal4 activation domain (AD) and DNA binding domain (DB), respectively, to proteins of interest [18]. pMW2 and pMW3 are reporter vectors for Y1H assays [49]. pMW2 is used to clone DNA fragments upstream of a HIS3 reporter gene, pMW3 is used to clone DNA fragments upstream of a LacZ reporter. pETG10A is used to generate N-terminal 6×His fusions for bacterial expression. pETG41A is used to generate N-terminal 6×His-MBP fusions for bacterial expression. pDEST-CMV-Myc is used to generate N-terminal Myc fusions under the control of a CMV promoter for mammalian cell expression. pKA921 is used for two-fragment Gateway cloning to create promoter-cDNA combinations. A polycistronic mCherry cassette with an unc-54 3′-UTR marks the tissues where the array is expressed. pCFJ150 is used to generate C. elegans expression vectors through three-fragment Gateway cloning [51]. pAG415 GAL-ccbB is used for constitutive expression of cDNAs in yeast [50]. pET-DUET1 (Novagen) is used for simultaneous expression of two cDNAs in bacteria. (DOCX) [file pgen.1003992.s010.docx]

**Table S3. Plasmids generated for this study.**

| **Plasmid** | **Parent plasmid** | **Notes** |
| --- | --- | --- |
| pJW93 | pDONR221 | FLAG-NHR-25 cDNA (with stop codon) entry clone |
| pJW109 | pDONR221 | SMO-1 cDNA entry clone |
| pJW121 | pAD-dest | SMO-1 AD fusion for Y2H |
| pJW135 | pAD-dest | NHR-25 full lenth (stopless)-AD fusion for Y2H |
| pJW136 | pDB-dest | NHR-25 full lenth (stopless)-DB fusion for Y2H |
| pJW165 | pDONR221 | NHR-91 cDNA entry clone |
| pJW238 | pDONR221 | NHR-25 L32F cDNA entry clone |
| pJW302 | pDONR221 | SMO-1 V31K cDNA entry clone |
| pJW305 | pDONR221 | NHR-25 K165R cDNA entry clone |
| pJW306 | pDONR221 | NHR-25 K170R cDNA entry clone |
| pJW309 | pDONR221 | SMO-1ΔGG cDNA entry clone |
| pJW310 | pAD-dest | SMO-1ΔGG in pAD vector for Y2H |
| pJW311 | pAD-dest | SMO-1 V31K in pAD vector for Y2H |
| pJW314 | pDONR221 | NHR-25 K165R K170R cDNA entry clone |
| pJW315 | pDONR221 | SMO-1ΔF cDNA (ends in GG) entry clone |
| pJW319 | pDB-dest | NHR-25 K165R in pDB vector for Y2H |
| pJW320 | pDB-dest | NHR-25 K170R in pDB vector for Y2H |
| pJW400 | pDONR-P4P1r | egl-17 promoter entry clone |
| pJW414 | pDB-dest | NHR-25 3KR in pDB vector for Y2H |
| pJW421 | pDONR221 | Myc-NHR-25 cDNA entry clone |
| pJW426 | pDB-dest | NHR-25 K236R in pDB vector for Y2H |
| pJW427 | pDONR221 | NHR-25 K236R, K165R cDNA entry clone |
| pJW428 | pDB-dest | NHR-25 K236R, K170R in pDB vector for Y2H |
| pJW432 | pDEST-CMV-Myc | NHR-25 N-terminal Myc fusion vector for CMV driven mammalian expression |
| pJW501 | pDB-dest | NHR-25 K236R, K165R in pDB vector for Y2H |
| pJW522 | pKA921 | P_egl-17:Myc-NHR-25 in polycistronic mCherry vector |
| pJW524 | pKA921 | P_wrt-2:Myc-NHR-25 in polycistronic mCherry vector |
| pJW526 | pKA921 | P_grl-21:Myc-NHR-25 in polycistronic mCherry vector |
| pJW630 | pDONR221 | NHR-25 3KR cDNA entry clone |
| pJW646 | pDONR221 | Myc-NHR-25 3KR cDNA entry vector |
| pJW647 | pETG-10A | 6xHIS-SMO-1 bacterial expression vector |
| pJW651 | pDONR201 | UBC-9 cDNA entry clone |
| pJW655 | pAD-dest | UBC-9 in pAD vector for Y2H |
| pJW656 | pDB-dest | UBC-9 in pDB vector for Y2H |
| pJW714 | pDONR221 | FLAG-NHR-25 (amino acids 161-541) cDNA entry clone |
| pJW722 | pETG-41A | 6xHIS-MBP-FLAG-NHR-25 (amino acids 161-541) bacterial expression vector |
| pJW741 | pDONR221 | TEV-UBC-9 cDNA entry clone |
| pJW742 | pDONR221 | TEV-SMO-1ΔF cDNA entry clone |
| pJW744 | pETG-10A | 6xHIS-TEV-UBC-9 bacterial expression vector |
| pJW746 | pETG-10A | 6xHIS-TEV-SMO-1ΔF bacterial expression vector |
| pJW751 | pET-DUET-1 | Ce E1 (6xHIS-TEV-Uba2 and native AOS-1) |
| pJW768 | pETG-41A | 6xHIS-MBP-FLAG-NHR-25 K170R, K263R (amino acids 161-541) bacterial expression vector |
| pJW773 | pKA921 | P_egl-17:Myc-SMO-1 in polycistronic mCherry vector for *C. elegans* expression |
| pJW774 | pKA921 | P_egl-17:Myc-NHR-25 3KR in polycistronic mCherry vector for *C. elegans* expression |
| pJW775 | pKA921 | P_grl-21:Myc-SMO-1 in polycistronic mCherry vector for *C. elegans* expression |
| pJW776 | pKA921 | P_wrt-2:Myc-SMO-1 in polycistronic mCherry vector for *C. elegans* expression |
| pJW782 | pETG-41A | 6xHIS-MBP-FLAG-NHR-25 3KR cDNA (amino acids 161-541) bacterial expression vector |
| pJW951 | pDONR221 | FLAG-NHR-25 3EA cDNA (amino acids 161-541) entry clone |
| pJW964 | pETG41A | 6xHIS-MBP-FLAG-NHR-25 3EA cDNA (amino acids 161-541) bacterial expression vector |
| pJW1027 | pDONR-P4P1r | 8xNR5RE(WT)-pes-10 minimal promoter with start codon entry clone |
| pJW1028 | pDONR-P4P1r | 8xNR5RE(MUT)-pes-10 minimal promoter with start codon entry clone |
| pJW1041 | pDONR221 | NLS-3xVenus entry clone (with artifical introns in Venus) |
| pJW1058 | pMW2 | 8xNR5RE(WT)-pes-10 minimal promoter driving Y1H HIS3 reporter |
| pJW1059 | pMW3 | 8xNR5RE(WT)-pes-10 minimal promoter driving Y1H LacZ reporter |
| pJW1060 | pMW2 | 8xNR5RE(MUT)-pes-10 minimal promoter driving Y1H HIS3 reporter |
| pJW1061 | pMW3 | 8xNR5RE(MUT)-pes-10 minimal promoter driving Y1H LacZ reporter |
| pJW1066 | pDEST-CMV-Myc | NHR-25 3KR in mammalian N-terminal Myc tagging vector |
| pJW1077 | pDONR221 | NHR-25 cDNA (amino acids 1-173) entry clone |
| pJW1084 | pAG415 GAL-ccbB | SMO-1ΔF, untagged, constitutive GPD promoter, LEU2 marker, CEN low copy origin |
| pJW1088 | pDONR221 | NHR-25 cDNA (with stop codon) entry clone |
| pJW1090 | pETG-41A | 6xHIS-MBP-NHR-25 cDNA (amino acids 1-173) cDNA bacterial expression vector |
| pJW1102 | pDONR-P4P1r | 2x MIS binding site entry clone |
| pJW1103 | pDONR-P4P1r | 2x MIS Mutated binding site entry clone |
| pJW1104 | pDONR-P4P1r | 2x CYP11A1 binding site entry clone |
| pJW1109 | pCFJ150 | 8xNR5RE(WT)-pes-10 minimal promoter::NLS-3xVenus::unc-54 3’-UTR *C. elegans* expression vector |
| pJW1110 | pCFJ150 | 8xNR5RE(MUT)-pes-10 min promoter::NLS-3xVenus::unc-54 3’-UTR *C. elegans* expression vector |
| pJW1115 | pAD-dest | NHR-25 L32F cDNA in pAD for Y2H |
| pJW1120 | pMW2 | 2x MIS driving Y1H HIS3 reporter |
| pJW1121 | pMW2 | 2x MIS Mutated driving Y1H HIS3 reporter |
| pJW1122 | pMW2 | 2x CYP11A1 driving Y1H HIS3 reporter |
| pJW1124 | pMW3 | 2x MIS driving Y1H LacZ reporter |
| pJW1125 | pMW3 | 2x MIS Mutated driving Y1H LacZ reporter |
| pJW1126 | pMW3 | 2x Cyp11A1 driving Y1H LacZ reporter |
